# Suppression of interferon α and γ response by Huwe1-mediated Miz1 degradation promotes SARS-CoV-2 replication

**DOI:** 10.3389/fimmu.2024.1388517

**Published:** 2024-07-05

**Authors:** Vinothini Arunagiri, Laura Cooper, Huali Dong, Jake Class, Indrani Biswas, Sujan Vahora, Riddhi Deshpande, Khushi H. Gopani, Guochang Hu, Justin M. Richner, Lijun Rong, Jing Liu

**Affiliations:** ^1^ Department of Surgery, College of Medicine, Cancer Center, University of Illinois at Chicago, Chicago, IL, United States; ^2^ Department of Microbiology and Immunology, College of Medicine, University of Illinois at Chicago, Chicago, IL, United States; ^3^ Departments of Anesthesiology and Pharmacology & Regenerative Medicine, College of Medicine, University of Illinois at Chicago, Chicago, IL, United States

**Keywords:** SARS-CoV-2, interferon alpha and gamma responses, Miz1, HUWE1, transcriptional regulation

## Abstract

Severe Acute Respiratory Syndrome Coronavirus 2 (SARS-CoV-2) has been demonstrated to limit the host interferon response; however, the underlying mechanism remains unclear. Here, we found that SARS-CoV-2 infection upregulated the E3 ubiquitin ligase Huwe1, which in turn facilitated the degradation of the transcription factor Miz1. The degradation of Miz1 hampered interferon alpha and gamma responses, consequently fostering viral replication and impeding viral clearance. Conversely, silencing or inhibiting Huwe1 enhanced the interferon responses, effectively curbing viral replication. Consistently, overexpressing Miz1 augmented the interferon responses and limited viral replication, whereas silencing Miz1 had the opposite effect. Targeting Huwe1 or overexpressing Miz1 elicited transcriptomic alterations characterized by enriched functions associated with bolstered antiviral response and diminished virus replication. Further study revealed Miz1 exerted epigenetic control over the transcription of specific interferon signaling molecules, which acted as common upstream regulators responsible for the observed transcriptomic changes following Huwe1 or Miz1 targeting. These findings underscore the critical role of the Huwe1-Miz1 axis in governing the host antiviral response, with its dysregulation contributing to the impaired interferon response observed during COVID-19.

## Introduction

Severe Acute Respiratory Syndrome Coronavirus 2 (SARS-CoV-2) is responsible for the ongoing coronavirus disease 2019 (COVID-19) pandemic, which has exerted substantial global impacts on both public health and the economy (1, 2). Despite the advancement of vaccines and antiviral drugs, the virus remains a formidable threat due to its propensity for mutation and the emergence of new variants (3). Consequently, a better understanding of the mechanisms underlying SARS-CoV-2 pathogenesis and host immunity is imperative for the development of more efficacious therapeutic interventions and control strategies.

A crucial facet of the host immune response to viral infections centers on the interferon (IFN) response. IFNs are a group of cytokines that play a critical role in the innate immune response against viral infections by inducing the expression of various interferon-stimulated genes (ISGs). These ISGs actively participate in various antiviral defense mechanisms, including viral recognition, clearance, and immunomodulation. However, emerging evidence has shown that SARS-CoV-2 has developed strategies to circumvent and suppress the host IFN response, thereby curtailing IFN production and signaling (4–10).

Several molecular mechanisms have been proposed to elucidate the limited IFN response during SARS-CoV-2 infection (10). One theory posits that the virus directly targets and inhibits critical components of the IFN signaling pathway. For example, the papain-like protease of SARS-CoV-2 (PLpro), an indispensable viral enzyme necessary for processing viral polyproteins to generate a functional replicase complex and enable viral spread (11, 12), has been shown to attenuate the IFN response. This attenuation occurs through direct cleavage of IFN regulatory factor 3 (IRF3) or indirect inhibition via deISGylation or deubiquitination (13–15). Additionally, PLpro dysregulates the stimulator of interferon genes (STING) by deubiquitination (16), deISGylates melanoma differentiation-associated protein 5 (MDA5) (17), or deubiquitinates components of the retinoic acid-inducible gene I (RIG-I)-like receptors (RLRs) signaling pathway, including RIG-I, mitochondrial antiviral signaling protein (MAVS), TANK-binding kinase 1 (TBK1), TNF receptor-associated factor 3 (TRAF3), and TRAF6 (15, 18). Another hypothesis suggests that the virus interferes with the production and secretion of IFNs themselves, possibly by targeting IFN-producing cells or disrupting the trafficking of IFNs to the cell surface (6, 7). However, the specific mechanisms responsible for the restricted IFN response during SARS-CoV-2 infection remain incompletely elucidated.

Myc-interacting zinc finger protein 1 (Miz1), also known as zinc finger and BTB domain-containing protein 17 (Zbtb17), is a member of the poxvirus and zinc-finger (POZ) domain/zinc finger transcription factor family. It contains an amino-terminal POZ domain, which is essential for its transcriptional activity, and 13 zinc fingers at its carboxyl terminus (19, 20). Miz1 preferentially binds at the initiation region of a gene and can either activate gene transcription directly or repress gene transcription by interacting with other regulatory factors, such as Myc, Myc associated factor X (Max), and B-cell lymphoma 6 protein (BCL-6) (21). Miz1 plays critical roles in cell proliferation, differentiation, cell-cycle progression, and apoptosis (22–24). Moreover, recent studies have indicated that Miz1 also participates in the regulation of the immune response (25–28), including negative regulation of the interferon signaling pathway during tumorigenesis (27, 28). However, its specific contribution to the antiviral response remains unclear.

In a previous study, we identified the HECT, UBA, and WWE domain-containing protein 1 (Huwe1), also known as Mcl-1 ubiquitin ligase E3 (Mule), URE-binding protein 1 (Ureb1), Large structure of UREB1 (LASU1), ARF-binding protein 1 (ARF-BP1), or Homologous to E6AP carboxyl terminus homologous protein 9 (HectH9), as the E3 ubiquitin ligase responsible for targeting Miz1 (29). Huwe1 is a highly conserved member of the HECT E3 ubiquitin ligase family and is involved in the ubiquitination and degradation of various proteins, including Histone H2A (30), p53 (31), N-Myc (32), Mcl-1 (33) and Cdc6 (34). It contains two Armadillo (ARM) repeat-like domains, a ubiquitin-associated (UBA) domain, a WWE domain, and a well-conserved BH3 domain at its N terminus, as well as a catalytic HECT domain at its C terminus (29, 33, 35, 36). Huwe1 serves as a sizable regulatory factor involved in numerous cellular processes, including cell cycle, DNA damage response, and apoptosis. However, the specific role of HUWE1 in the antiviral immune response to SARS-CoV-2 is currently unknown.

In this study, we aimed to investigate the molecular mechanisms responsible for the restricted IFN response observed during SARS-CoV-2 infection. We found that dysregulation of the Huwe1-Miz1 axis contributed to diminished IFN response in COVID-19. Significantly, our findings suggest that targeting either Huwe1 or Miz1 could potentially bolster host immune responses and enhance viral clearance. These insights offer a fresh perspective on the molecular mechanisms underlying SARS-CoV-2 pathogenesis and host immunity, which may pave the way for the development of innovative therapies and control strategies for managing COVID-19.

## Results

### SARS-CoV-2 infection upregulates Huwe1 leading to degradation of Miz1

**Figure 1:**
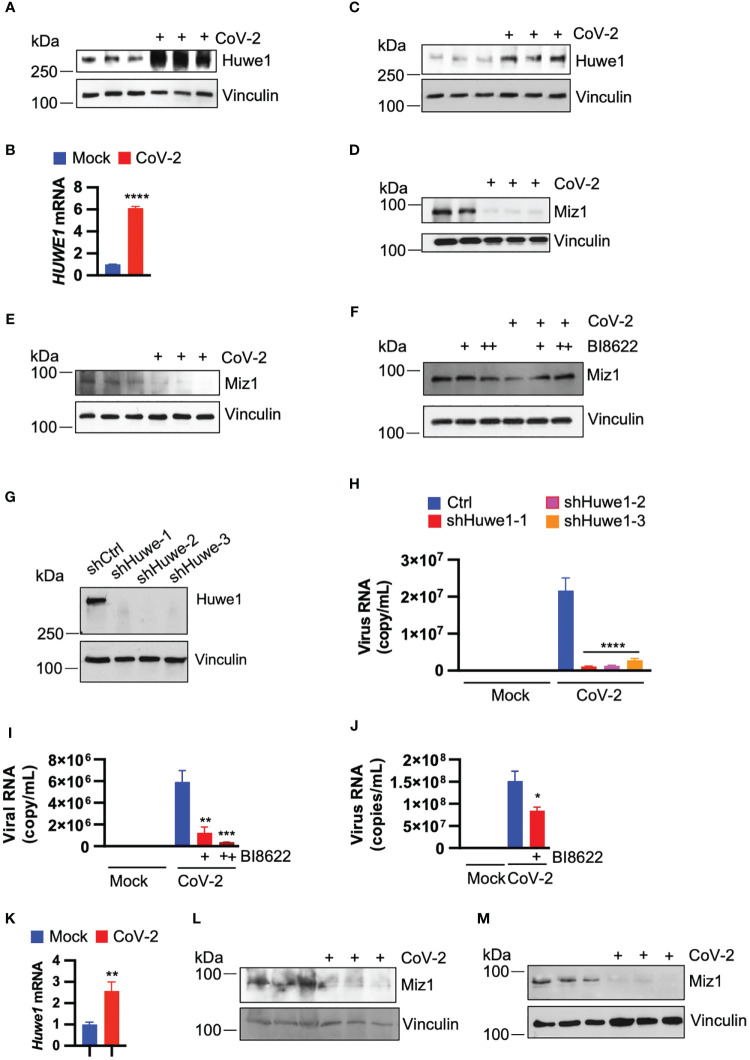
SARS-CoV-2 infection upregulates Huwe1 leading to the degradation of Miz1 protein. **(A)** Huwe1 protein expression and **(B)** Huwe1 mRNA expression in mock- or SARS-CoV-2 infected A549/hACE2 cells at 24 h post-infection. **(C)** Huwe1 protein expression in mock- or SARS-CoV-2 infected Vero E6 cells at 24 h post-infection. Miz1 protein expression in mock- or SARS-CoV-2 infected **(D)** A549/hACE2 cell and **(E)** Vero E6 cells at 24 h post-infection. **(F)** Miz1 protein expression in mock- or SARS-CoV-2 infected A549/hACE2 cells without or with BI8622 at 24 h post-infection. **(G)** Huwe1 protein expression in A549/hACE2 cells expressing shRNAs targeting Huwe1 at three different sites. **(H)** Viral titers in the cell culture supernatants from mock- or SARS-CoV-2 infected A549/hACE2 cells expressing shRNAs targeting Huwe1 at three different sites at 48 h post-infection. **(I)** Viral titers in the cell culture supernatants from mock- or SARS-CoV-2 infected A549/hACE2 cells without or with different doses of BI8622 at 48 h post-infection. +, 10 μM; ++, 20 μM. **(J)** Viral titers in the cell culture supernatants from mock- or SARS-CoV-2 infected Vero E6 cells without or with BI8622 at 48 h post-infection. **(K)** Huwe1 mRNA expression in untreated or SARS-CoV-2 infected K18-hACE2 mice. **(L, M)** Miz1 protein expression in untreated or SARS-CoV-2 infected K18-hACE2 mice at 2 d post-infection **(L)** or BALB/c mice at 3 d post-infection **(M)**. In **(B, H–J, K)**, values represent the mean ± SEM. n=3. Unpaired Student’s *t*-test was used. *p < 0.05; **p < 0.01; ***p < 0.001; ****p < 0.0001.

To investigate the effect of SARS-CoV-2 infection on the expression of Huwe1, we inoculated A549/hACE2 cells (a human lung epithelial cell line that overexpresses the SARS-CoV-2 receptor, human angiotensin converting enzyme 2 (hACE2) under the control of human cytomegalovirus immediate early promoter) with SARS-CoV-2 (USA/WA1/2020). We observed an increase in both Huwe1 protein and mRNA expression at 24 h post-infection (**Figures 1A, B**). Similarly, we found an increase in Huwe1 expression in SARS-CoV-2-infected Vero E6 cells (**Figure 1C**), a monkey kidney epithelial cell line that is highly susceptible to SARS-CoV-2 (37). We previously reported that Huwe1 targets Miz1 for ubiquitination and proteasomal degradation (29). Accordingly, we observed a decrease in Miz1 protein levels in SARS-CoV-2-infected A549/hACE2 or Vero E6 cells (**Figures 1D, E**). We also found that Miz1 mRNA expression was indeed increased after SARS-CoV-2 infection in A549/hACE2 and Vero E6 cells (**Supplementary Figures 1A, B**), further supporting our conclusion that the decrease in Miz1 protein levels is a result of post-translational regulation. The upregulation of Miz1 mRNA expression is likely a feedback response to the downregulation of the protein. A specific small molecule inhibitor of Huwe1, BI8622 (MedChemExpress), has been developed, and our previous studies have demonstrated that it effectively inhibits Huwe1 activity, resulting in the stabilization of Miz1. We found that BI8622 prevented SARS-CoV-2-induced degradation of Miz1 (**Figure 1F**). Taken together, these findings suggest that SARS-CoV-2 infection upregulates Huwe1, which leads to the degradation of Miz1.

As SARS-CoV-2 infection leads to dysregulation of Huwe1, we explored whether Huwe1 affects the replication of SARS-CoV-2. To do so, we generated stable A549/hACE2 cells expressing lentiviral small hairpin RNAs (shRNAs) targeting Huwe1 at three different sites (**Figure 1G**). Our results showed that the silencing of Huwe1 significantly reduced viral titers in the cell culture supernatants at 48 h post-infection with SARS-CoV-2 (**Figure 1H**). We also found that the inhibition of Huwe1 using BI8622 in A549/hACE2 and Vero E6 cells led to similar outcomes (**Figures 1I, J**).

To ascertain whether the regulatory patterns of Huwe1 and Miz1 that we observed *in vitro* are likewise evident *in vivo*, we administered SARS-CoV-2 (WA1) to K18-hACE2 mice. These mice are genetically engineered to express the human ACE2 protein under the control of the keratin 18 promoter, leading to its expression in epithelial cells. In line with the findings from our cell culture experiments, SARS-CoV-2 infection resulted in an increase in Huwe1 mRNA expression and a decrease in Miz1 protein levels in the lungs of K18-hACE2 mice (**Figures 1K, L**). Similarly, a reduction in Miz1 protein expression was also observed in BALB/c mice infected with the mouse-adapted SARS-CoV-2 MA10 (**Figure 1M**).

### Silencing of Huwe1 augments the interferon signaling during SARS-CoV-2 infection

**Figure 2:**
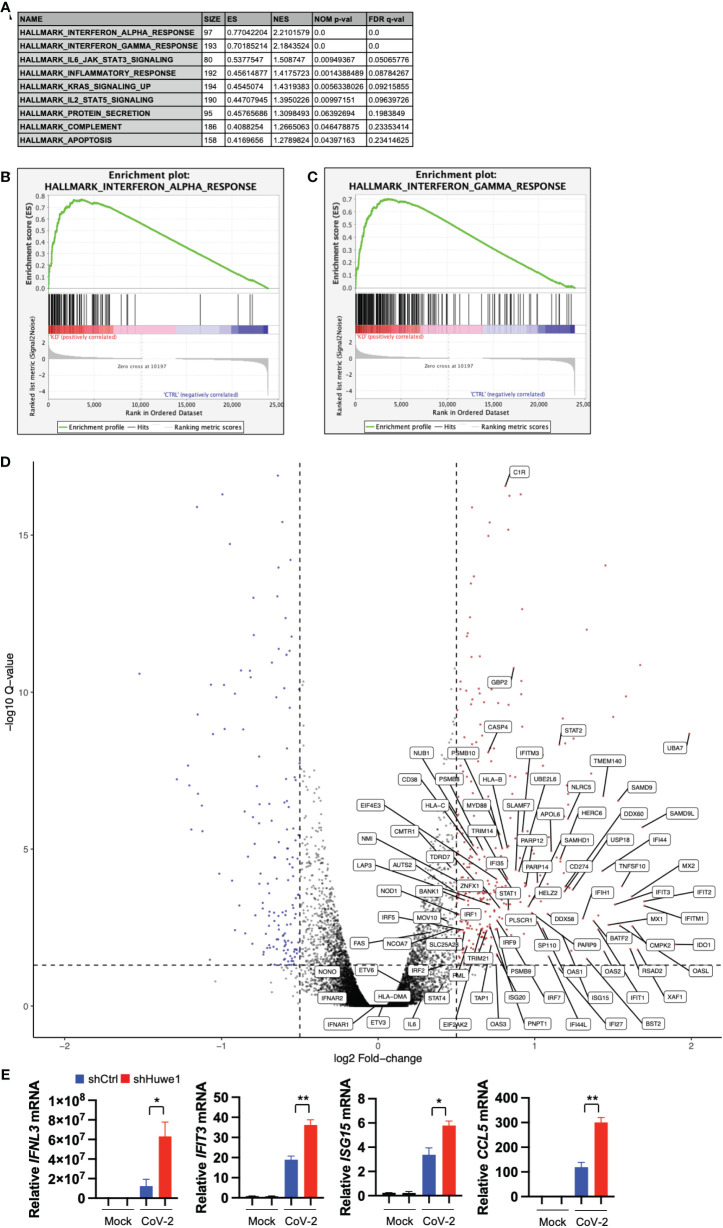
Silencing Huwe1 augments interferon signaling during SARS-CoV-2 infection. **(A)** GSEA showing enriched gene sets (FDR <0.25) by Huwe1 KD in SARS-CoV-2-infected A549/hACE2 cells at 24 h post-infection. **(B, C)** GSEA showing enrichment plots of the gene sets of “Interferon alpha response” **(B)** and “Interferon gamma response” **(C)** by Huwe1 KD in SARS-CoV-2 infected A549/hACE2 cells at 24 h post-infection. **(D)** Volcano plots showing fold change and p-values of the top differentially expressed genes enriched in the interferon pathways by Huwe1 KD in SARS-CoV-2 infected A549/hACE2 cells at 24 h post-infection. The horizontal line represents the q-value threshold of 0.05, while vertical lines indicate log fold change (log_2_FC) thresholds at 0.5 and -0.5. **(E)** mRNA expression of IFNL3, IFIT3, ISG15, and CCL5 by Huwe1 KD in mock- or SARS-CoV-2-infected A549/hACE2 cells at 24 h post-infection as analyzed by qRT-PCR. Values represent the mean ± SEM. n=3. Unpaired Student’s *t*-test was used. *p < 0.05; **p < 0.01.

To gain insight into the mechanism by which Huwe1 promotes SARS-CoV-2 replication, we conducted RNA-seq analysis on both control and Huwe1 knock-down (KD) A549/hACE2 cells to explore the impact of Huwe1 on the transcriptomics of SARS-CoV-2 infection. Gene set enrichment analysis (GSEA) of differentially expressed genes identified interferon alpha and gamma responses as the top two significantly enriched gene sets in the Huwe1 KD group compared to the control group in response to SARS-CoV-2 infection (false discovery rate (FDR) <0.25, **Figures 2A–C**). Note, normalized enrichment score (NES): 2.210 for interferon alpha response and 2.184 for interferon gamma response (**Figure 2A**). Enrichment plots of interferon alpha response (**Figure 2B**) and interferon gamma response (**Figure 2C**) show significant upregulation in the Huwe1 KD group compared to control group during virus infection. Silencing of Huwe1 upregulated various molecules involved in interferon alpha and gamma signaling, such as interferon regulatory factors (IRFs), interferon-stimulated genes (ISGs), human leukocyte antigens (HLAs) in major histocompatibility complexes (MHC), and signal transducer and activator of transcription (STATs), among others (**Figure 2D**, **Supplementary Figures 2A, B**). Additionally, we observed upregulated gene sets of inflammatory response and interleukin (IL) 6 signaling in the Huwe1 KD group compared to the control group during SARS-CoV-2 infection (**Figures 2A** **,** **Supplementary Figures 2C–F**). While an exuberant inflammatory response, also known as cytokine storm, can contribute to severe COVID-19, a well-regulated and controlled inflammatory response coordinates the recruitment of specific subsets of leukocytes involved in the antiviral response, helping to clear the virus (38). Therefore, the enhanced inflammatory response and IL6 signaling, in addition to the upregulated interferon signaling, may contribute to the observed increased viral clearance by Huwe1 KD (**Figure 1H**). It’s worth noting that the RNA-seq findings were validated by qRT-PCR analysis of randomly selected genes, including interferon lambda-1 (IFNL3), interferon-induced protein with tetratricopeptide repeats 3 (IFIT3), interferon-stimulated gene 15 (ISG15), and C-C motif chemokine ligand 5 (CCL5) (**Figure 2E**).

**Figure 3:**
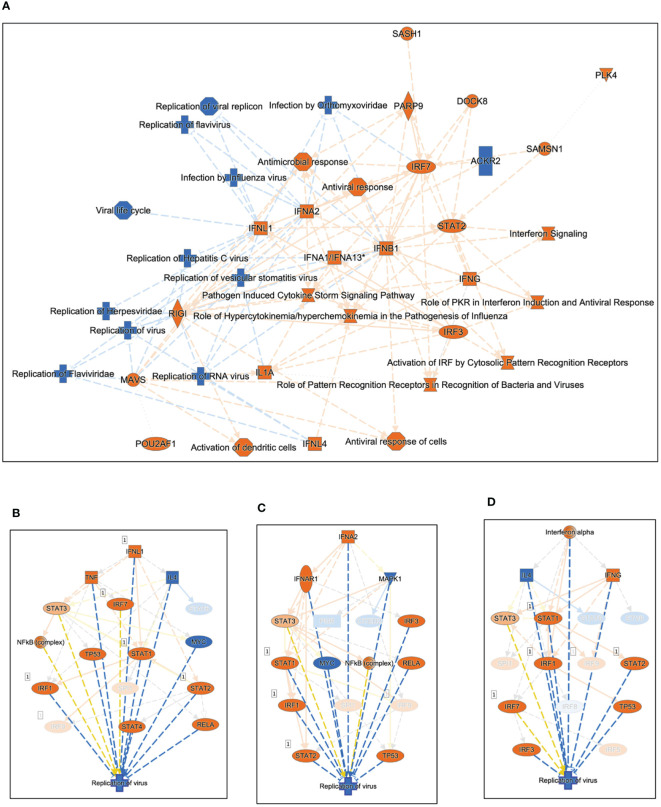
Silencing Huwe1 enhances the antiviral response during SARS-CoV-2 infection. **(A)** Using IPA core analysis, a visual representation of the most significant canonical pathways and biological networks resulting from Huwe1 KD in A549/hACE2 cells at 24 h post SARS-CoV-2 infection revealed interferon signaling and antiviral response. IPA upstream analysis revealed **(B)** IFNL1, **(C)** IFNA2, and **(D)** Interferon alpha gene cluster among the top upstream regulators responsible for the gene expression changes by Huwe1 KD in A549/hACE2 cells at 24 h post SARS-CoV-2 infection.

We used Ingenuity Pathway Analysis (IPA) to investigate the relationship between highly significant genes (adjusted *P* value <0.05 and absolute fold-change ≥2) in SARS-CoV-2-infected Huwe1 KD cells compared to virus-infected control cells. Our analysis revealed the most significant canonical pathways and biological networks linked to interferon signaling and antiviral response (**Figure 3A**), which is consistent with the results of GSEA (**Figures 2A–C**). Note that in the visualization, upregulation is depicted by orange nodes, while downregulation is represented by blue nodes. It is evident that Huwe1 KD leads to upregulation of interferon signaling and antiviral response pathways, alongside downregulation of viral replication pathways (**Figure 3A**). To predict the key upstream regulators responsible for the observed changes in gene expression by Huwe1 KD during SARS-CoV-2 infection, we performed IPA upstream functional analysis. This analysis is based on the literature compiled in the Ingenuity Pathway Knowledge Base (IPKB). The analysis examined the presence of known targets of the upstream regulators among those highly significant genes by Huwe1 KD during SARS-CoV-2 infection and computed an overlap *P* value based on significant overlap between genes in our dataset and known targets regulated by the regulator. The activation z-score algorithm was used to make predictions. Our IPA analysis identified several top upstream regulators that were activated in our dataset and are known to play a role in the antiviral response. These include interferon lambda-1 (IFNL1; Activation Z-score: 7.125; *p*=5.6E-69), interferon alpha gene cluster (Activation Z-score: 7.949; *p*=1.68E-60), interferon alpha-2 (IFNA2; Activation Z-score: 8.314; *p*=2.18E-60), interferon regulatory factor 7 (IRF7; Activation Z-score: 7.797; *p*=7.49E-58), signal transducer and activator of transcription 1 (STAT1; Activation Z-score: 7.018; *p*=1.15E-57), and Non-POU domain-containing octamer-binding protein (NONO; Activation Z-score: 8.314; *p*=2.18E-60) (**Figures 3B–D** **,** **Supplementary Table 1**). Previous studies have shown that these proteins are involved in various cellular processes such as DNA repair, RNA processing, and gene regulation, and have been reported to play a role in antiviral responses (39–46). IPA also predicted top upstream regulators that were inhibited in our dataset, such as three prime repair exonuclease 1 (TREX1; Activation Z-score: -6.205; *p*= 1.19E-63), ETS Variant Transcription Factor 3 (ETV3; Activation Z-score: -6.782; *p*=1.27E-62) and ETV6 (Activation Z-score: -6.608; *p*=2.46E-56; **Supplementary Table 1**), which have been reported to inhibit IFN-stimulated genes (47, 48). In IPA, causal networks are generated by predicting the upstream regulators that are most likely responsible for the changes in gene expression observed in a given experimental dataset. These upstream regulators are inferred from prior knowledge in IPKB, and the downstream targets of these regulators are analyzed to identify key regulatory nodes that drive the observed changes in gene expression. Using this approach, Causal Network Analysis in IPA identified several important regulatory nodes in the resulting causal networks, including IFNL1, IFNA2, interferon-α/β receptor (IFNAR), ETV3, IRF7, NONO, and ETV6 (**Supplementary Figures 3A–D**, **Supplementary Table 2**). Finally, Using the Diseases and Functions Analysis feature in IPA, the submitted experimental dataset was analyzed to predict the most relevant biological functions and pathways by comparing the input gene list to the IPA Knowledge Base. The analysis revealed decreased coronavirus replication function (Z-score=-2.798; overlap p-value=1.07E-12; **Supplementary Table 3**), consistent with the observed decrease in viral replication caused by Huwe1 KD during SARS-CoV-2 infection (**Figures 1H, I**).

### Inhibition of Huwe1 using the BI8622 inhibitor recapitulates the effects of Huwe1 knockdown during SARS-CoV-2 infection

**Figure 4:**
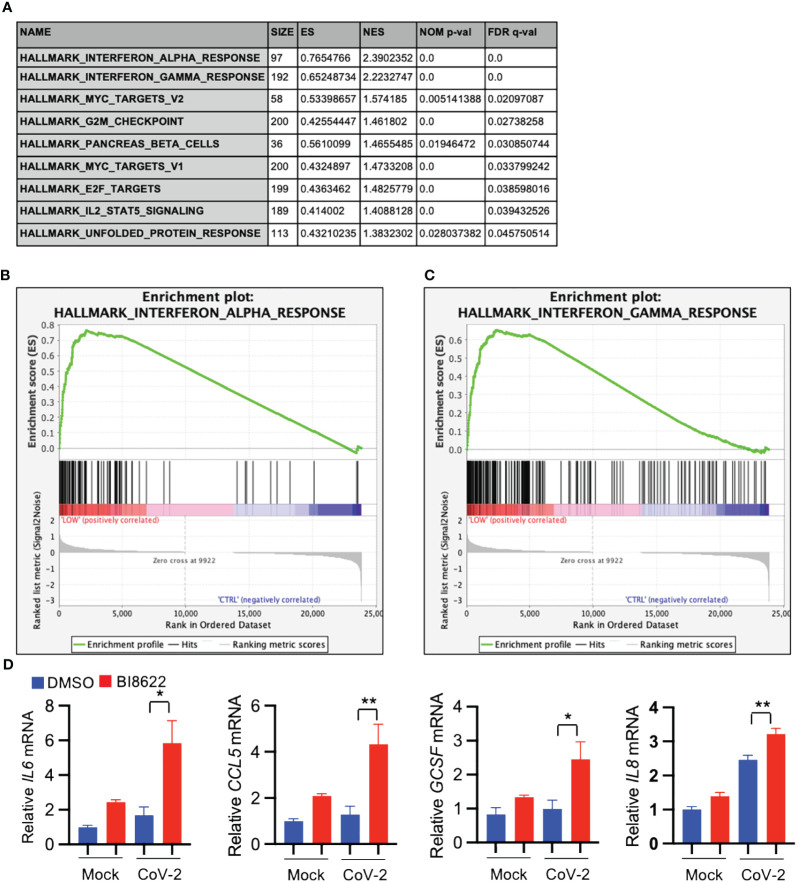
Inhibition of Huwe1 with BI8622 recapitulates the effects of HUWE1 silencing on interferon signaling and antiviral response during SARS-CoV-2 infection. **(A)** GSEA showing enriched gene sets (FDR <0.25) by inhibition of Huwe1 with BI8622 in SARS-CoV-2 infected A549/hACE2 cells at 24 h post-infection. **(B, C)** GSEA showing enrichment plots of the gene sets of “Interferon alpha response” and “Interferon gamma response” by inhibition of Huwe1 with BI8622 in SARS-CoV-2-infected A549/hACE2 cells at 24 h post-infection. **(D)** mRNA expression of IL6, CCL5, GCSF, and IL8 by inhibition of Huwe1 with BI8622 in mock- or SARS-CoV-2-infected A549/hACE2 cells at 24 h post-infection. Values represent the mean ± SEM. N=3. Unpaired Student’s *t*-test was used. *p < 0.05; **p < 0.01.

GSEA analysis of differentially expressed genes revealed that inhibition of Huwe1 using the BI8622 inhibitor resulted in an enhanced interferon response to SARS-CoV-2 infection, similar to the effect observed with Huwe1 KD (**Figures 4A–C**). The RNA-seq results were validated by qRT-PCR of randomly selected genes, including IL6, CCL5, GCSF, and IL8 (**Figure 4D**). Additionally, IPA analysis identified the most significant canonical pathways and biological networks associated with interferon signaling and antiviral response (**Supplementary Figure 4A**), along with top upstream regulators, such as IFNL1, TREX1, NONO, interferon alpha gene cluster, ETV3, and ETV6 (**Supplementary Figures 4B, C**, **Supplementary Table 4**). Causal Network Analysis in IPA further revealed crucial regulatory nodes, including interferon alpha or IFNAR gene cluster, IFNL1, NONO, and ETV3 (**Supplementary Figures 4D–F**, **Supplementary Table 5**). Furthermore, Diseases and Functions Analysis in IPA predicted a decrease in virus replication (Z-score=-2.851; overlap p-value=5.99E-20; **Supplementary Table 6**). These results are consistent with the effects observed from Huwe1 KD. Overall, these findings suggest that BI8622-mediated inhibition of Huwe1 has a similar effect to Huwe1 KD in enhancing the interferon response to SARS-CoV-2 infection.

### Miz1 overexpression enhances antiviral response against SARS-CoV-2 infection

**Figure 5:**
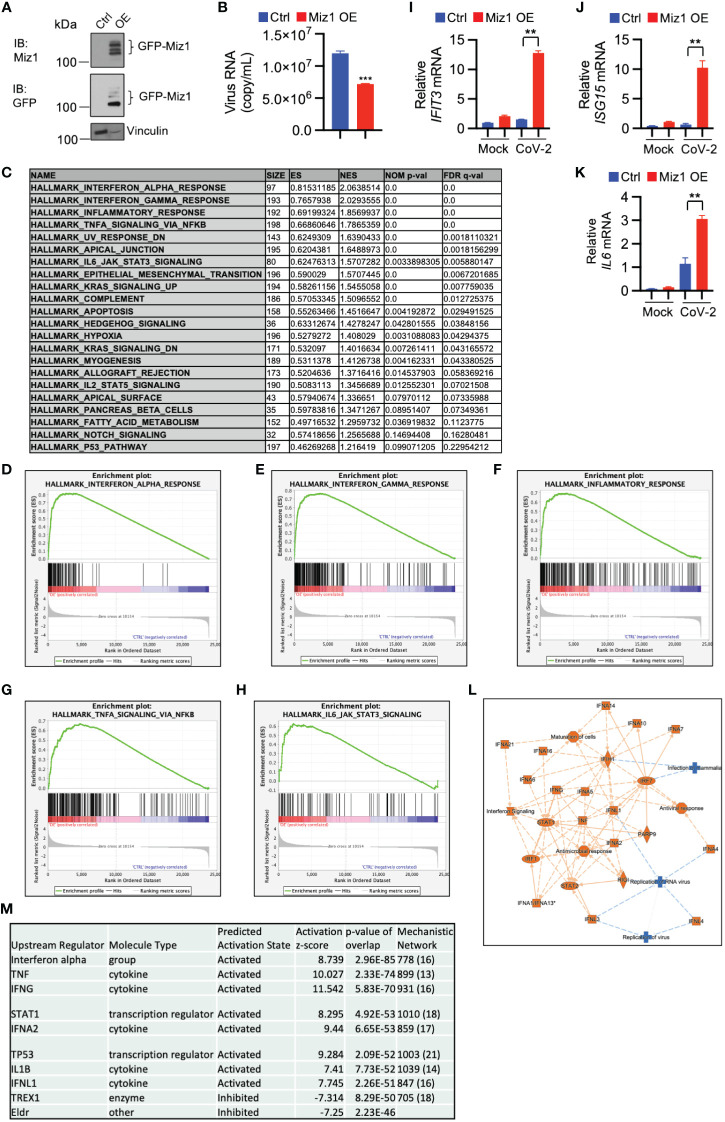
Miz1 overexpression enhances interferon signaling and antiviral response during SARS-CoV-2 infection. **(A)** Western blot analysis of exogenous GFP-Miz1 in stable A549/hACE2 cells overexpressing lentiviral GFP-tagged Miz1 (Miz1 OE cells). **(B)** Viral titers in the cell culture supernatants from SARS-CoV-2-treated A549/hACE2 cells expressing control lentiviral vector or lentiviral vector containing GFP-Miz1 at 48 h post infection. **(C)** GSEA showing enriched gene sets (FDR <0.25) by Miz1 overexpression in SARS-CoV-2-infected A549/hACE2 cells at 24 h post infection. **(D-H)** GSEA showing enrichment plots of the gene sets of “Interferon alpha response”, “Interferon gamma response”, “Inflammatory response”, “TNF signaling via NF-κB”, and “IL6 signaling” by Miz1 overexpression in SARS-CoV-2-infected A549/hACE2 cells at 24 h post infection. **(I-K)** mRNA expression of IFIT3, ISG15, and IL6 in mock- or SARS-CoV-2-infected control and Miz1 OE cells at 24 h post infection. **(L)** IPA core analysis identified interferon signaling and antiviral response as the most significant canonical pathways and biological networks affected by Miz1 overexpression in SARS-CoV-2-infected A549/hACE2 cells at 24 h post infection. **(M)** IPA upstream analysis revealed the top ten upstream regulators responsible for the gene expression changes by Miz1 overexpression in SARS-CoV-2-infected A549/hACE2 cells at 24 h post infection. In **(B, I-K)**, Values represent the mean ± SEM. n=3. Unpaired Student’s *t*-test was used. **p < 0.01; ***p < 0.001.

Our data indicate that inhibition or knockdown of Huwe1 enhances the antiviral response during SARS-CoV-2 infection. This phenomenon is likely attributed to the upregulation of Miz1, as Huwe1 targets Miz1 for degradation. To test this hypothesis, we generated stable A549/hACE2 cells that overexpressed exogenous green fluorescence protein (GFP)-tagged Miz1 using a lentiviral vector (**Figure 5A**). We found that Miz1 overexpression (OE) resulted in decreased viral titers in the cell culture supernatants at 48 hours post-infection with SARS-CoV-2 (**Figure 5B**). These findings align with the results obtained from the experiments involving Huwe1 KD or Huwe1 inhibition.

Furthermore, the GSEA of RNA-seq data from control or Miz1 overexpressing cells treated with SARS-CoV-2 showed enriched functions in interferon alpha and gamma, inflammatory response, TNF, and IL6 signaling by Miz1 OE (**Figures 5C–H**). The RNA-seq results were validated by qRT-PCR of randomly selected genes, including IFIT3, ISG15, and IL6 (**Figures 5I–K**). Additionally, IPA revealed significant canonical pathways and biological networks involved in interferon signaling and antiviral response (**Figure 5L**), with molecules such as interferon alpha gene cluster, STAT1, IFNA2, IFNL1, and TREX1 identified as top upstream regulators (**Figure 5M**). These findings suggest that Miz1 overexpression mimics the effects of Huwe1 KD or Huwe1 inhibition in enhancing the antiviral response to SARS-CoV-2 infection.

### Knockdown of Miz1 impairs antiviral response to SARS-CoV-2 infection

**Figure 6:**
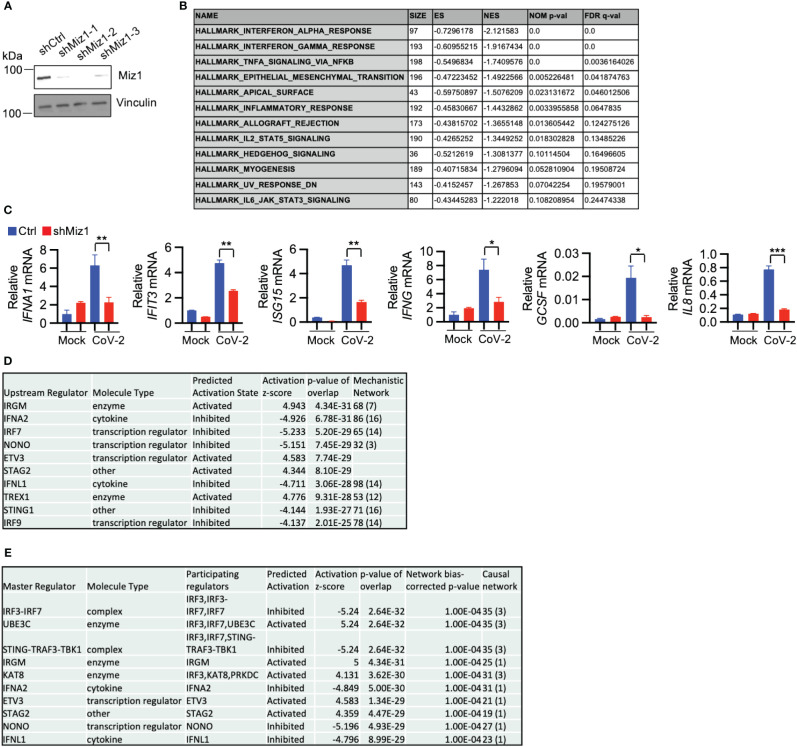
Miz1 silencing suppresses interferon response during SARS-CoV-2 infection. **(A)** Protein expression of Miz1 in stable A549/hACE2 cells expressing shRNAs targeting Miz1 at three different sites. **(B)** GSEA showing downregulated enriched gene sets (FDR <0.25) in SARS-CoV-2-infected A549/hACE2 cells with Miz1 KD at 24 h post infection. **(C)** mRNA expression of IFNA1, IFIT3, ISG15, IFNG, GCSF, IL8 in mock- or SARS-CoV-2-infected A549/hACE2 cells with Miz1 KD at 24 h post infection. Values represent the mean ± SEM. N=3. Unpaired Student’s *t*-test was used. *p < 0.05; **p < 0.01; ***p < 0.001. **(D)** IPA upstream analysis revealing the top ten upstream regulators responsible for the gene expression changes by Miz1 KD in SARS-CoV-2-infected A549/hACE2 cells at 24 h post infection. **(E)** Causal Network Analysis in IPA identifying the top ten master regulators in the resulting causal networks.

In contrast to the effects of Miz1 OE, Miz1 KD reduces interferon alpha and gamma response, TNF signaling, inflammatory response, and IL-6 signaling during SARS-CoV-2 infection, as demonstrated by the GSEA of RNA-seq data from A549/hACE2 cells that stably express lentiviral shRNA for Miz1 (**Figures 6A, B**, **Supplementary Figures 5A–E**). The RNA-seq results were validated by qRT-PCR of randomly selected genes, including interferon alpha 1 (IFNA1), IFIT3, ISG15, interferon gamma (IFNG), granulocyte colony-stimulating factor (GCSF), and interleukin-8 (IL8) (**Figure 6C**). Furthermore, IPA analysis identified significant canonical pathways and biological networks involved in inhibited interferon signaling and antiviral response by Miz1 KD (**Supplementary Figure 5F**). Upstream regulator analysis identified IRF7, IFNL1, IFNA2, NONO, and ETV3 as top upstream regulators inhibited by Miz1 KD (**Figure 6D**) and present in the causal networks (**Figure 6E**). Notably, these molecules are also observed as top upstream regulators and in the causal networks in the case of Miz1 OE (**Figure 5M**) or Huwe1 KD (**Supplementary Tables 1**, **2**) or inhibition (**Supplementary Tables 4**, **5**), but with an opposite activation direction.

**Figure 7:**
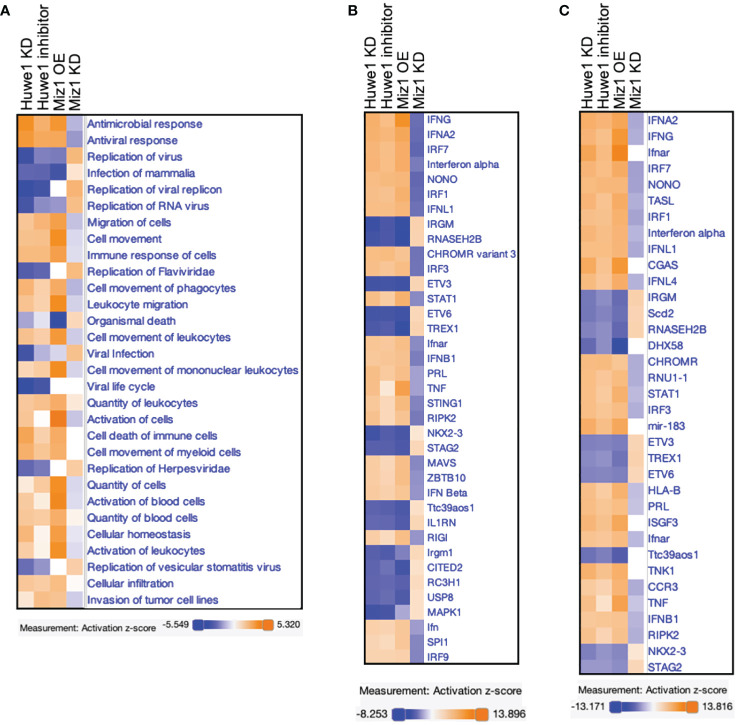
Comparison analysis of RNA-seq data from Huwe1 KD or inhibitor, Miz1 OE, and Miz1 KD during SARS-CoV-2 infection using IPA. **(A)** Diseases and Functions Analysis of the RNA-seq data showing the top biological functions and diseases affected by Huwe1 KD or inhibition, Miz1 OE, and Miz1 KD in SARS-CoV-2-infected A549/hACE2 cells at 24 h post infection. **(B)** Upstream Regulator Analysis of the RNA-seq data showing the top upstream regulators responsible for the gene expression changes in each condition. **(C)** Causal Network Analysis of the RNA-seq data showing the top master regulators and their interactions in the resulting causal networks for each condition.

Finally, The RNA-seq data from Huwe1 KD or inhibitor, Miz1 OE, and Miz1 KD were subjected to IPA analysis for a parallel comparison. The Diseases and Functions Analysis revealed the most relevant biological functions and pathways related to enhanced antimicrobial and antiviral responses with reduced virus replication by Huwe1 KD or inhibitor or Miz1 OE. Conversely, Miz1 KD had the opposite effects (**Figure 7A**). The upstream regulator analysis showed that IFNA2, interferon alpha gene cluster, and IFNL1 were among the top upstream regulators (**Figure 7B**) and were present in the causal networks (**Figure 7C**). These regulators were activated by Huwe1 KD or inhibitor and Miz1 OE but inhibited by Miz1 KD (**Figures 7B, C**). Taken together, these data suggest that Huwe1 KD or inhibition phenocopies the effects of Miz1 OE, which can be reversed by Miz1 KD. These findings imply that Huwe1 plays a role in evading and dampening the host antiviral response during SARS-CoV-2 infection by downregulating Miz1.

### Miz1 epigenetically regulates the interferon signaling molecules

**Figure 8:**
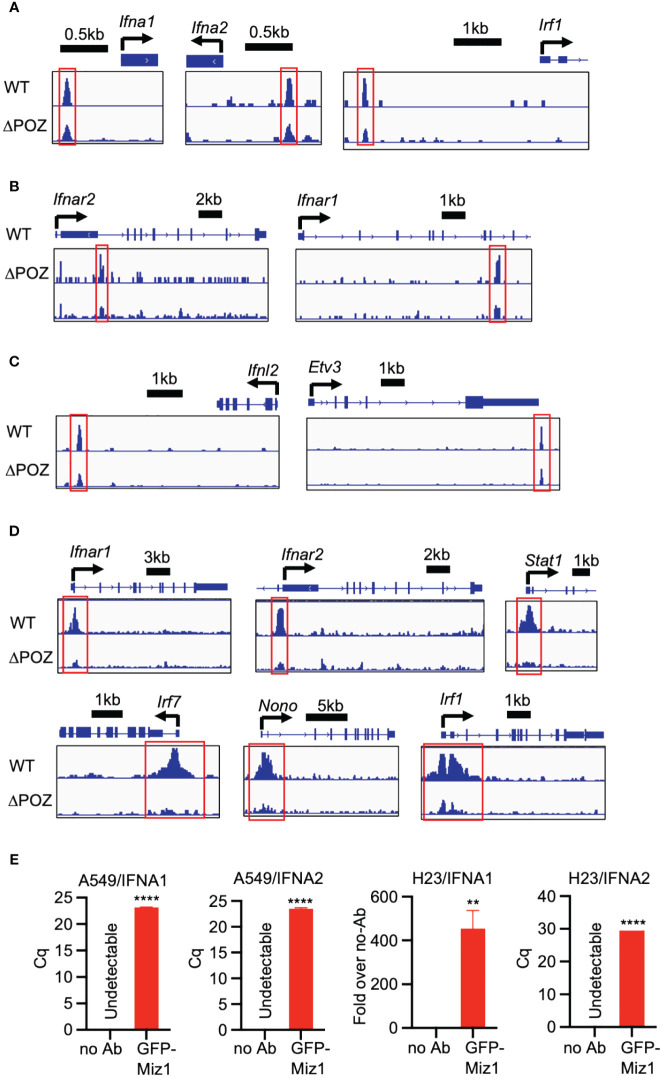
Miz1 epigenetically regulates interferon signaling molecules. **(A-C)** ChIP-seq tracks showing Miz1 binding on the promoter region **(A)**, intronic region **(B)**, or downstream **(C)** of the interferon signaling molecules as indicated. **(D)** ChIP-seq tracks showing H3K9/14ac on the promoter region of the interferon signaling molecules in MLE-12/Miz1(WT) and MLE-12/Miz1(ΔPOZ) as indicated. **(E)** ChIP-qPCR showing enrichment of Miz1 binding on the promoters of *IFNA1* and *IFNA2* in comparison to the no antibody (no Ab) control in A549 and NCI-H23 cells stably expressing GFP-Miz1 as indicated. Values represent the mean ± SEM. N=3. Unpaired Student’s *t*-test was used. **p < 0.01; ****p < 0.0001. In cases where the “no Ab” control yielded undetectable results, quantification cycle (Cq) values are presented instead of “Fold over no-Ab”.

**Figure 9:**
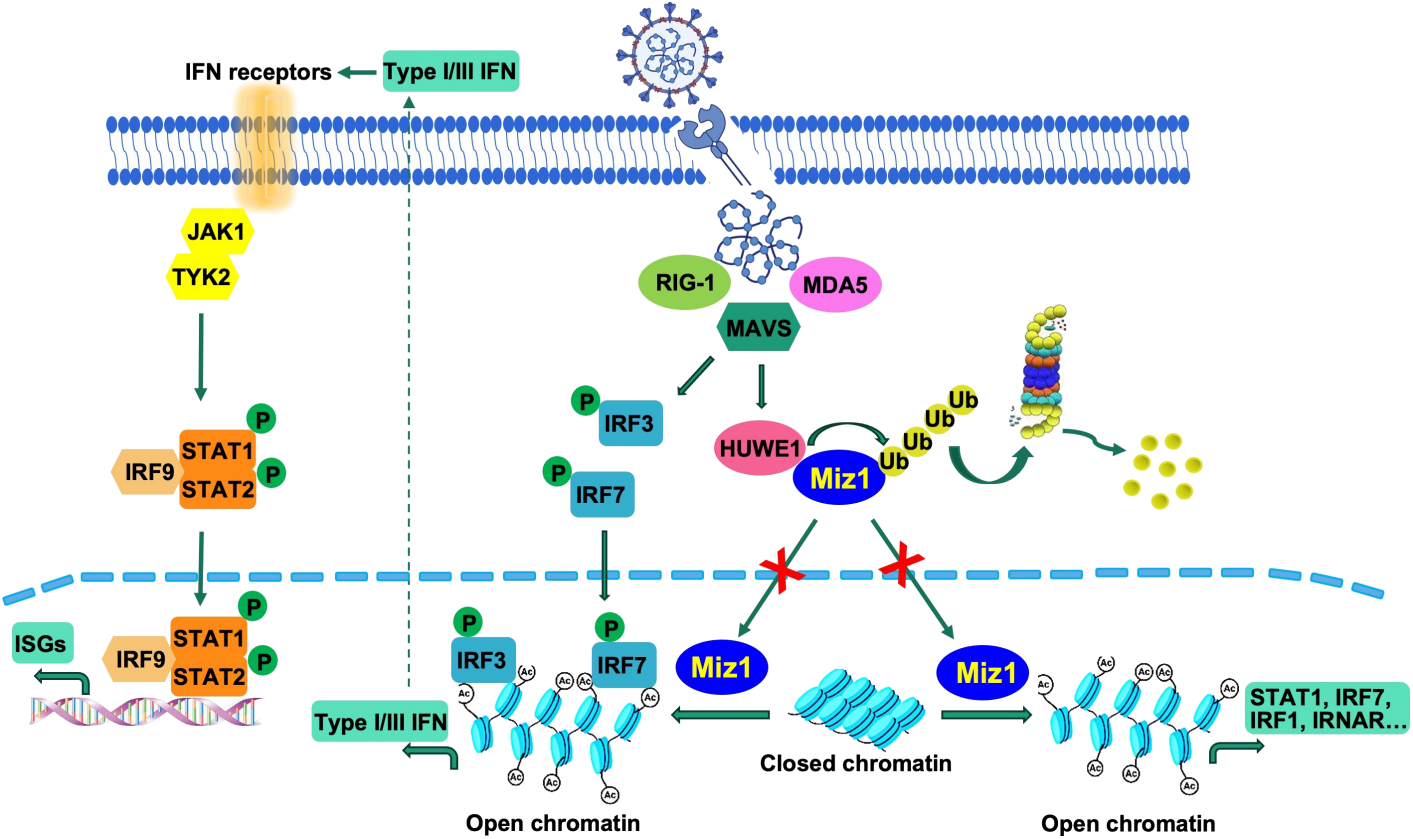
Illustration of suppression of the interferon response by Huwe1-mediated degradation of Miz1. Upon SARS-CoV-2 infection, the RIG-I pathway activates the downstream IRF3/7 pathways. Concurrently, Huwe1 is upregulated, resulting in the ubiquitination and degradation of Miz1. In the absence of degradation, Miz1 facilitates the epigenetic upregulation of interferon signaling molecules through histone acetylation, effectively inhibiting viral replication.

Transcription factors regulate gene expression by binding to various regions of the DNA surrounding the target gene, including the proximal promoter region upstream of the transcription start site, as well as regions within introns, exons, and downstream regions of the gene. To understand the mechanism by which Miz1 regulates interferon signaling molecules, we queried our data from chromatin immunoprecipitation followed by sequencing (ChIP-seq) in stable murine type II-like lung epithelial cells (MLE-12) expressing shRNA against endogenous Miz1 and exogenous shRNA-resistant Miz1 mutant with the POZ domain deletion (ΔPOZ) or wild-type (WT) Miz1, as we previously reported (26). Our Miz1 ChIP-seq analysis revealed that Miz1 binds to the regulatory elements of certain interferon signaling molecules that are identified as upstream regulators of Miz1 or Huwe1 targeting. Notably, Miz1 was found to bind to the proximal promoter region upstream of the transcription start site of *Ifna1*, *Ifna2*, and *Irf1* (**Figure 8A**), the intronic region of *Ifnar2* and *Ifnar1* (**Figure 8B**), and downstream of *Ifnl2* (**Figure 8C**). The POZ domain deletion reduced Miz1 binding to these regulatory elements (**Figures 8A–C**). Depending on binding partners and cellular context, Miz1 can mediate transcriptional activation or repression (20, 49–51). We have previously reported that Miz1 is involved in epigenetic regulation through histone deacetylation (26, 52, 53). Histone acetylation is a crucial post-translational modification of histones that regulates chromatin organization and gene expression (54). Histone acetylation, catalyzed by histone acetyltransferases (HATs), increases accessibility of transcription factors to DNA, resulting in transcriptional activation. Conversely, histone deacetylation, catalyzed by histone deacetylases (HDACs), has the opposite effect and is generally associated with transcriptional repression (54). Our ChIP-seq analysis revealed increased histone H3 acetyl lysine 9 or 14 (H3K9/14ac), which are representative acetylation marks associated with active promoters, gene expression, and transcription factor binding (55, 56), on the promoter region of the upstream regulators of Miz1 or Huwe1 targeting, including *Ifnar1*, *Ifnar2*, *Irf1*, *Irf7*, *Stat1*, and *Nono* in Miz1(WT) cells as compared to Miz1(ΔPOZ) cells (**Figure 8D**). These findings indicate that Miz1 plays a role in driving the increased transcription of these genes. These data are consistent with predicted activation of these molecules by Miz1 OE and Huwe1 KD or inhibition, while predicted inhibition of these molecules by Miz1 KD (**Figure 7B**). We further validated Miz1 binding on the promoter region of *IFNA1* and *IFNA2* in A549 or human lung epithelial NCI-H23 cells, which stably express exogenous green fluorescent protein (GFP)-tagged Miz1 as we previously reported (52, 53) (**Figure 8E**). Overall, these results suggest that Miz1 epigenetically regulates interferon signaling molecules by directly or indirectly binding to target genes and regulating histone acetylation. Taken together, our data underscores the crucial role of the Huwe1-Miz1 axis in orchestrating the host antiviral response during SARS-CoV-2 infection (**Figure 9**).

## Discussion

The COVID-19 pandemic has left an indelible mark on global public health, posing a formidable challenge to healthcare systems worldwide. The immune system plays a crucial role in controlling SARS-CoV-2 infection, and its dysregulation is associated with disease severity. The interferon response is a critical component of the innate immune response that controls viral replication and spread. However, SARS-CoV-2 has been shown to limit the host interferon response, but the underlying mechanism is not fully understood.

In our study, we aimed to investigate the mechanism underlying the limited host interferon response during SARS-CoV-2 infection. Our results revealed that SARS-CoV-2 infection increases the expression of the E3 ubiquitin ligase Huwe1, which then targets its substrate, the transcription factor Miz1, for degradation. This degradation of Miz1 results in the inhibition of interferon alpha and gamma response, which promotes viral replication and hampers viral clearance. Our study highlights the crucial role of the Huwe1-Miz1 axis in regulating the host antiviral response and indicates its dysregulation as a potential contributor to the limited interferon response during COVID-19. The upregulation of Huwe1 is likely to be a result of viral manipulation of the host’s machinery to evade the host immune response. Our findings offer new mechanistic insights into how SARS-CoV-2 can evade the host immune response by suppressing interferon signaling. Our study adds to the growing body of evidence indicating that the host immune response plays a critical role in SARS-CoV-2 pathogenesis.

Our study identified several common upstream regulators enriched in interferon signaling molecules through IPA analysis of Huwe1 or Miz1 targeting, including interferon alpha or IFNAR gene cluster, IFNL1, IFNA2, IRF1, IRF7, STAT1, NONO, TREX1, ETV3, and ETV6. Targeting these upstream regulators could potentially enhance the host antiviral response and reduce virus replication, highlighting their potential as therapeutic targets. Furthermore, our study found that Miz1 regulates the expression of some of these genes through histone acetylation, by binding to their regulatory elements or other mechanisms. Specifically, Miz1 increases histone acetylation on the promoter regions of interferon signaling molecules, leading to their transcriptional activation. In the current study, Miz1 activates the transcription of interferon signaling molecules by increasing histone acetylation on their promoter regions. Our previous research has shown that Miz1 represses angiotensin converting enzyme 2 (Ace2), protocadherin 10 (Pcdh10), interleukin 12A (Il12a), and cyclin-dependent kinase inhibitor 1A (Cdkn1a, p21 gene) by inducing histone deacetylation through the recruitment of histone deacetylase 1 (HDAC1) (26, 52, 53). These findings highlight the versatility of Miz1 in regulating gene expression through different binding partners and cellular contexts.

Our findings suggest that targeting the Huwe1-Miz1 axis could serve as a potential therapeutic approach to bolster the host antiviral response and reduce virus replication. The Huwe1 specific inhibitor BI8622 could represent a viable therapeutic approach to enhance interferon response and limit viral replication. In addition, the overexpression of Miz1 or the inhibition of its degradation may represent an alternative approach to amplify interferon signaling and promote viral clearance. Our study provides a foundation for future investigations into the development of antiviral therapies targeting the Huwe1-Miz1 axis.

This study only focused on the role of Huwe1 and Miz1 in the immune response to SARS-CoV-2 infection, and future studies may need to investigate their potential roles in other aspects of the infection, such as viral replication and pathogenesis. Other factors, such as viral proteins or host immune molecules, may also contribute to the dysregulation of the interferon response. Future studies should aim to investigate the interaction between the Huwe1-Miz1 axis and other host-virus factors in regulating the immune response to SARS-CoV-2 and the results need to be validated in animal models and human samples. Our previous report indicated that Miz1 suppressed ACE2 transcription through epigenetic repression of the ACE2 promoter. However, in the present study, we utilized A549/hACE2 cells, which exhibit an overexpression of exogenous hACE2 not controlled by the endogenous physiological promoter. Consequently, the regulatory impact and functional implications of Miz1 on ACE2 are minimal in this context. In other words, the regulatory influence of Miz1 on the immune response to SARS-CoV-2 infection appears to be independent of its role in ACE2 expression regulation.

In conclusion, our study provides new insights into the molecular mechanisms underlying SARS-CoV-2 evasion of the host immune response. We have identified a novel mechanism by which the virus upregulates Huwe1, leading to the degradation of Miz1 and inhibition of interferon alpha and gamma response, promoting viral replication and hindering viral clearance. Targeting the Huwe1-Miz1 axis could provide a promising approach to developing new antiviral therapies against COVID-19.

## Methods

### Mice

We utilized 6–8-week-old K18-hACE2 mice in our study. These mice are genetically engineered to express the human ACE2 protein under the control of the keratin 18 promoter, thereby directing its expression in epithelial cells. These mice were procured from Jackson Laboratories (Stock No: 034860). We also used BALB/c mice (JAX; Strain #:000651).

### SARS-CoV-2 source

The SARS-Related Coronavirus 2, Isolate USA-WA1/2020 (NR-52281) was provided by the Centers for Disease Control and Prevention and acquired through BEI Resources, NIAID, NIH. The SARS-Related Coronavirus 2 Mouse Adapted (MA10) virus was obtained from the Baric Lab at UNC. The viruses were cultured and quantified on Vero-E6 cells (ATCC). In brief, Vero cells were maintained in DMEM with 10% Fetal Bovine Serum (FBS) and Glutamax, with cells having fewer than 20 passages used for all experiments. Virus stocks were amplified in Vero-E6 cells after initial inoculation with a low MOI (0.01) and harvested four days later. Viral titers were determined using a plaque assay on Vero-E6 cells. To prevent genetic drift, viral stocks were used after a single expansion (passage = 1).

### SARS-CoV-2 infection of mice

K18-hACE2 mice and BALB/c mice were anesthetized with isoflurane and intranasally challenged with 5x10^4^ PFU of the SARS-CoV-2 WA1 or MA10 respectively. For intranasal infection, mice were inoculated with the virus in a 50 µL droplet administered to both the left and right nostrils of each mouse. Inhalation of the droplet was confirmed for each mouse. Mouse infections were conducted at the University of Illinois at Chicago (UIC) in accordance with biosafety level 3 (BSL3) guidelines. All animal care and experimental procedures were conducted in compliance with institutional and U.S. National Institutes of Health (NIH) guidelines and were approved by the UIC Institutional Animal Care and Use Committee (IACUC).

At 2-, 3-, and 5-dpi, tissues were harvested and homogenized in PBS. To isolate protein, tissue homogenates were prepared with 1% triton X-100 and incubated for one hour at room temperature for inactivation. RNA was isolated using the RNeasy Mini Kit (Qiagen Cat# 74104) protocol and eluted in a volume of 40 μL of nuclease free water.

### SARS-CoV-2 infection of cells

Cells were inoculated with SARS-CoV-2 (USA/WA1/2020; BEI Resources) at MOI (multiplicity of infection) = 0.5, and at 2 h, virus-containing media were removed and cells were washed with PBS to remove unbound virus particles before being replenished with fresh media. At 24 or 48 h, cell culture supernatants were collected for viral quantification, and cells were lysed for Western Blot or RNA-seq. All SARS-CoV-2 assays were performed in the BSL-3 facility at UIC.

### Viral quantification

The viral RNA was extracted using TRIzol (ThermoFisher) and RNA purification was performed using Invitrogen™ PureLink™ RNA Mini Kit (12183025, Invitrogen). Viral titers were quantified by one step qRT-PCR using TaqMan™ Fast Virus 1-Step Master Mix (Applied Biosystems™ 4444432) and SARS-CoV-2 RUO qPCR Primer & Probe Kit (10006713, IDT).

### Antibodies for western blot

Miz-1 Antibody (D7E8B, Cell Signaling Technology; 1:500), β-actin antibody (A5441, Sigma-Aldrich; 1:20,000), Huwe1 antibody (ab70161, abcam; 1:500), Vinculin antibody (MA5–11690, Invitrogen; 1;500), GFP antibody (3E6, ThermoFisher; 1:500) were used for Western Blot.

### Stable KD cell lines

Stable Miz1 KD cell lines were generated in A549/hACE2 cells with SMARTvector Lentiviral shRNAs targeting human Miz1 (V3SVHS02_8619648 with target sequence AGTTCACGCACACGGGGAA, V3SVHS02_4756503 with target sequence CGGCCCTTCTGACTGTTTA, V3SVHS02_10078083 with target sequence AGCGCTGCGGCAAGAGATT; Horizon Discovery) or non-targeting control (Cat #S02–005000–01; Horizon Discovery) according to the manufacturer’s instructions and selected with puromycin (0.5 μ g/mL). Stable Huwe1 KD cell lines were generated in A549/hACE2 cells with SMARTvector Lentiviral shRNAs targeting human Huwe1 (V3SVHS09_5138611 with target sequence AGCATTGCGTTCTTTCGAT, V3SVHS09_6205369 with target sequence TTGGTGAGGTCCTATGTTG, V3SVHS09_7079440 with target sequence CCAGCTAGGCATTCGTCCA; Horizon Discovery) according to the manufacturer’s instructions and selected with puromycin (0.5 μ g/mL).

### RNA-seq

The RNA-seq was conducted by the University of Chicago Genomics Facility. Briefly, Total RNA was extracted and purified using NucleoSpin RNA kit. The quality and quantity of RNA were evaluated using the Agilent Bio-analyzer. The generation of stranded oligo-dT-based NGS libraries was performed using the Illumina stranded mRNA library kit. Indexed sequence libraries were pooled for multiplexing, and paired-end sequencing (100 bp) was carried out on NovaSEQ6000 with dual-index sequencing primers (Illumina). The alignment of reads to the hg19 reference genome was performed using TopHat2, and the evaluation of differential expression was performed using edgeR, which was carried out by the Research Informatics Core at the Research Resources Center of the University of Illinois at Chicago. The enrichment analysis was performed using curated databases such as GSEA and IPA.

### GSEA analysis of differentially expressed genes

The GSEA analysis utilized version 4.2.2 of the GSEA software and the Molecular Signatures Database (MSigDB), which contains predefined gene sets sharing pathways, functions, chromosomal localization, or other features. Specifically, the H collection sets, which comprise hallmark gene sets in MSigDB, were used in this study. Gene sets containing 15 to 500 genes were selected based on minimum and maximum criteria, respectively. Significantly enriched gene sets were determined based on a false discovery rate (FDR) of less than 0.25.

### IPA

The list of differentially expressed genes targeting Huwe1 or Miz1, containing gene identifiers and corresponding expression values (log ratio), was uploaded into the IPA software (Qiagen). Subsequently, the differentially expressed data was analyzed using the “core analysis” function included in the software, which covered biological processes, canonical pathways, upstream regulators, and gene networks. The parameters for the core analysis were set to an adjusted P value of less than 0.05 and an absolute fold-change greater than or equal to 2. To identify significant pathways associated with the differentially expressed genes, a pathway analysis was performed. Furthermore, an upstream regulator analysis was conducted to identify regulators that may be responsible for the observed gene expression changes. A significance threshold of a p-value less than 0.05 and a predicted activation z-score greater than 2 or less than -2 was set for this analysis. To understand the relationships between genes and pathways, a causal network analysis was conducted. Moreover, a disease and function analysis was performed to identify the biological functions and diseases that were most significantly associated with the differentially expressed genes. Finally, a comparison analysis was performed using the “Compare Analysis” function in IPA to compare groups targeting Huwe1 and Miz1, and to identify the biological differences between them. This analysis utilizes various statistical methods such as t-tests, ANOVA, or non-parametric tests to identify the differentially expressed genes between the groups.

### Quantitative PCR, ChIP assay, and ChIP-seq

For quantitative PCR (qPCR), we used iQ™ SYBR^®^ Green Supermix (Bio-Rad) and a CFX Connect™ Real-Time PCR Detection System (Bio-Rad). To obtain mRNA expression levels of a specific gene, we normalized the data to hypoxanthine-guanine phosphoribosyltransferase (HPRT) and the primer sequences are provided in **Supplementary Table 7**. For qRT-PCR, total RNA was extracted from lung tissues or isolated cells using NucleoSpin RNA kit (Macherey-Nagel) and cDNA was synthesized with M-MuLV Reverse Transcriptase (NEB) following the manufacturer’s instructions.

For ChIP assays, cells were treated with 25 mM EGS for 20 min and then fixed with 1% formaldehyde for 10 min. 1 M glycine (dissolved in PBS) was used to quench the fixation. Cells were then lysed with ChIP SDS Lysis Buffer (Millipore, Catalog # 20–163) with protease inhibitors (100 mM PMSF, 10 mM PNPP, 1 mM Na_3_VO_4_, 1 mM DTT, 1 μg/ml aprotinin). Lysates were subjected to sonication on ice to shear DNA to fragments between 200 and 500 bp in size. Sonication efficiency was determined by analyzing samples by 1% agarose gel electrophoresis. Sonicated DNA (50 μg) was subjected to immunoprecipitation with the appropriate antibody (1 to 2 μg). Precipitated DNA-bead complexes were washed once with Low Salt Immune Complex Wash Buffer (Catalog # 20–154), once with High Salt Immune Complex Wash Buffer (Catalog # 20–155), and twice each with LiCl Immune Complex Wash Buffer (Catalog # 20–156) and TE Buffer (Catalog # 20–157). Eluted DNA was purified and subjected to qPCR with primers spanning the gene promoter regions (**Supplementary Table 8**).

ChIP-seq was performed and analyzed as we previously reported (53), using the following antibodies for immunoprecipitation: anti-Miz1 (H-190 X, Santa Cruz Biotechnology) and anti-acetyl-histone H3 (06–599; MilliporeSigma). Briefly, we quantitated DNA samples with Qubit and prepared libraries using Illumina TruSEQ, which were then sequenced on an Illumina NovaSEQ6000 (100 bp, paired-end) at the University of Chicago Genomics Facility. We demultiplexed raw sequencing data using Illumina bcl2fastq and aligned sequencing reads to the mouse genome mm10 using BWA MEM. To prevent PCR duplication artifacts from affecting downstream results, we removed PCR duplicates using Picard MarkDuplicates. We called ChIP peaks relative to inputs with Macs2, produced normalized bedgraph tracks with the –SPMR flag, and converted these to bigWig tracks using the UCSC tool bedGraphToBigWig.

### Statistical analysis

Statistical analysis was performed using an unpaired Student’s t-test assuming normal distribution and equal variance. Adequate power was ensured by selecting sample sizes based on preliminary results. A *p* value < 0.05 was considered statistically significant.

## Data availability statement

The original contributions presented in the study are publicly available. This data can be found here: https://figshare.com/s/a42d4625020f37ef40a6; https://figshare.com/s/12ed66649a9e4c35f9bb; https://figshare.com/s/b012e1289755401ec862; https://figshare.com/s/b56c7e7e8779d1be6d70.

## Ethics statement

The animal study was approved by UIC Institutional Animal Care and Use Committee (IACUC). The study was conducted in accordance with the local legislation and institutional requirements.

## Author contributions

VA: Formal analysis, Investigation, Validation, Writing – review & editing. LC: Formal analysis, Investigation, Validation, Writing – review & editing. HD: Formal analysis, Investigation, Validation, Writing – review & editing. JC: Formal analysis, Investigation, Validation, Writing – review & editing. IB: Formal analysis, Investigation, Validation, Writing – review & editing. SV: Formal analysis, Investigation, Validation, Writing – review & editing. RD: Formal analysis, Investigation, Validation, Writing – review & editing. KG: Formal analysis, Investigation, Validation, Writing – review & editing. GH: Conceptualization, Writing – review & editing. JR: Resources, Writing – review & editing. LR: Conceptualization, Resources, Supervision, Writing – review & editing. JL: Funding acquisition, Resources, Supervision, Writing – original draft, Writing – review & editing.

## References

[1] Lamers MM Haagmans BL. SARS-coV-2 pathogenesis. Nat Rev Microbiol. (2022) 20:270–84. doi: 10.1038/s41579-022-00713-0 35354968

[2] Zhu N Zhang D Wang W Li X Yang B Song J. A novel coronavirus from patients with pneumonia in China, 2019. N Engl J Med. (2020) 382:727–33. doi: 10.1056/NEJMoa2001017 PMC7092803 31978945

[3] Murakami N Hayden R Hills T Al-Samkari H Casey J Sorbo Del L. Therapeutic advances in COVID-19. Nat Rev Nephrol. (2023) 19:38–52. doi: 10.1038/s41581-022-00642-4 36253508 PMC9574806

[4] Blanco-Melo D Nilsson-Payant BE Liu WC Uhl S Hoagland D Møller R. Imbalanced host response to SARS-coV-2 drives development of COVID-19. Cell. (2020) 181:1036–1045.e9. doi: 10.1016/j.cell.2020.04.026 32416070 PMC7227586

[5] Acharya D Liu G Gack MU. Dysregulation of type I interferon responses in COVID-19. Nat Rev Immunol. (2020) 20:397–8. doi: 10.1038/s41577-020-0346-x PMC7249038 32457522

[6] Minkoff JM tenOever B. Innate immune evasion strategies of SARS-CoV-2. Nat Rev Microbiol. (2023) 21:178–94. doi: 10.1038/s41579-022-00839-1 PMC9838430 36631691

[7] Banerjee AK Blanco MR Bruce EA Honson DD Chen LM Chow A. SARS-coV-2 disrupts splicing, translation, and protein trafficking to suppress host defenses. Cell. (2020) 183:1325–1339.e21. doi: 10.1016/j.cell.2020.10.004 33080218 PMC7543886

[8] Miorin L Kehrer T Sanchez-Aparicio MT Zhang K Cohen P Patel RS. SARS-CoV-2 Orf6 hijacks Nup98 to block STAT nuclear import and antagonize interferon signaling. Proc Natl Acad Sci U.S.A. (2020) 117:28344–54. doi: 101073/pnas2016650117 10.1073/pnas.2016650117 PMC7668094 33097660

[9] Wong LR Perlman S. Immune dysregulation and immunopathology induced by SARS-CoV-2 and related coronaviruses - are we our own worst enemy? Nat Rev Immunol. (2022) 22:47–56. doi: 10.1038/s41577-021-00656-2 34837062 PMC8617551

[10] Hadjadj J Yatim N Barnabei L Corneau A Boussier J Smith N. Impaired type I interferon activity and inflammatory responses in severe COVID-19 patients. Science. (2020) 369:718–24. doi: 10.1126/science.abc6027 PMC7402632 32661059

[11] Harcourt BH Jukneliene D Kanjanahaluethai A Bechill J Severson KM Smith CM. Identification of severe acute respiratory syndrome coronavirus replicase products and characterization of papain-like protease activity. J Virol. (2004) 78:13600–12. doi: 10.1128/JVI.78.24.13600-13612.2004 PMC533933 15564471

[12] Lim KP Ng LF Liu DX. Identification of a novel cleavage activity of the first papain-like proteinase domain encoded by open reading frame 1a of the coronavirus Avian infectious bronchitis virus and characterization of the cleavage products. J Virol. (2000) 74:1674–85. doi: 10.1128/JVI.74.4.1674-1685.2000 PMC111642 10644337

[13] Moustaqil M Ollivier E Chiu HP Tol Van S Rudolffi-Soto P Stevens C. SARS-CoV-2 proteases PLpro and 3CLpro cleave IRF3 and critical modulators of inflammatory pathways (NLRP12 and TAB1): implications for disease presentation across species. Emerg Microbes Infect. (2021) 10:178–95. doi: 10.1080/22221751.2020.1870414 PMC7850364 33372854

[14] Shin D Mukherjee R Grewe D Bojkova D Baek K Bhattacharya A. Papain-like protease regulates SARS-CoV-2 viral spread and innate immunity. Nature. (2020) 587:657–62. doi: 10.1038/s41586-020-2601-5 PMC7116779 32726803

[15] Ran XH Zhu JW Chen YY Ni RZ Mu D. Papain-like protease of SARS-CoV-2 inhibits RLR signaling in a deubiquitination-dependent and deubiquitination-independent manner. Front Immunol. (2022) 13:947272. doi: 10.3389/fimmu.2022.947272 36032116 PMC9411789

[16] Cao D Duan L Huang B Xiong Y Zhang G Huang H. The SARS-CoV-2 papain-like protease suppresses type I interferon responses by deubiquitinating STING. Sci Signal. (2023) 16:eadd0082. doi: 10.1126/scisignal.add0082 37130168

[17] Liu G Lee JH Parker ZM Acharya D Chiang JJ Gent van M. ISG15-dependent Activation of the RNA Sensor MDA5 and its Antagonism by the SARS-CoV-2 papain-like protease. bioRxiv (2020). doi: 10.1101/2020.10.26.356048 PMC8103894 33727702

[18] Setaro AC Gaglia MM. All hands on deck: SARS-CoV-2 proteins that block early anti-viral interferon responses. Curr Res Virol Sci. (2021) 2:100015. doi: 10.1016/j.crviro.2021.100015 34786565 PMC8588586

[19] Wanzel M Herold S Eilers M. Transcriptional repression by myc. Trends Cell Biol. (2003) 13:146–50. doi: 10.1016/S0962-8924(03)00003-5 12628347

[20] Peukert K Staller P Schneider A Carmichael G Hänel F Eilers M. An alternative pathway for gene regulation by Myc. EMBO J. (1997) 16:5672–86. doi: 10.1093/emboj/16.18.5672 PMC1170199 9312026

[21] Riva L Yuan S Yin X Martin-Sancho L Matsunaga N Pache L. Discovery of SARS-CoV-2 antiviral drugs through large-scale compound repurposing. Nature. (2020) 586:113–9. doi: 10.1038/s41586-020-2577-1 PMC7603405 32707573

[22] Herold S Wanzel M Beuger V Frohme C Beul D Hillukkala T. Negative regulation of the mammalian UV response by Myc through association with Miz-1. Mol Cell. (2002) 10:509–21. doi: 10.1016/S1097-2765(02)00633-0 12408820

[23] Saito M Novak U Piovan E Basso K Sumazin P Schneider C. BCL6 suppression of BCL2 via Miz1 and its disruption in diffuse large B cell lymphoma. Proc Natl Acad Sci U.S.A. (2009) 106:11294–9. doi: 10.1073/pnas.0903854106 PMC2708681 19549844

[24] Si J Yu X Zhang Y DeWille JW. Myc interacts with Max and Miz1 to repress C/EBPdelta promoter activity and gene expression. Mol Cancer. (2010) 9:92. doi: 10.1186/1476-4598-9-92 20426839 PMC2879254

[25] Do-Umehara HC Chen C Zhang Q Misharin AV Abdala-Valencia H Casalino-Matsuda SM. Epithelial cell-specific loss of function of Miz1 causes a spontaneous COPD-like phenotype and up-regulates Ace2 expression in mice. Sci Adv. (2020) 6:eabb7238. doi: 10.1126/sciadv.abb7238 32851183 PMC7428331

[26] Do-Umehara HC Chen C Zhang Q Schleimer RP Budinger GRS Liu J. Suppression of allergic asthma by loss of function of miz1-mediated th1 skewing. Am J Respir Cell Mol Biol. (2022) 67:346–59. doi: 10.1165/rcmb.2022-0135OC PMC9447135 35833903

[27] Zimmerli D Brambillasca CS Talens F Bhin J Linstra R Romanens L. MYC promotes immune-suppression in triple-negative breast cancer via inhibition of interferon signaling. Nat Commun. (2022) 13:6579. doi: 10.1038/s41467-022-34000-6 36323660 PMC9630413

[28] Muthalagu N Monteverde T Raffo-Iraolagoitia X Wiesheu R Whyte D Hedley A. Repression of the type I interferon pathway underlies MYC- and KRAS-dependent evasion of NK and B cells in pancreatic ductal adenocarcinoma. Cancer Discovery. (2020) 10:872–87. doi: 10.1158/2159-8290.CD-19-0620 PMC7611248 32200350

[29] Yang Y Do H Tian X Zhang C Liu X Dada LA. E3 ubiquitin ligase Mule ubiquitinates Miz1 and is required for TNFalpha-induced JNK activation. Proc Natl Acad Sci U.S.A. (2010) 107:13444–9. doi: 10.1073/pnas.0913690107 PMC2922175 20624960

[30] Liu Z Oughtred R Wing SS. Characterization of E3Histone, a novel testis ubiquitin protein ligase which ubiquitinates histones. Mol Cell Biol. (2005) 25:2819–31. doi: 10.1128/MCB.25.7.2819-2831.2005 PMC1061639 15767685

[31] Chen D Kon N Li M Zhang W Qin J Gu W. ARF-BP1/Mule is a critical mediator of the ARF tumor suppressor. Cell. (2005) 121:1071–83. doi: 10.1016/j.cell.2005.03.037 15989956

[32] Zhao X Heng JI Guardavaccaro D Jiang R Pagano M Guillemot F. The HECT-domain ubiquitin ligase Huwe1 controls neural differentiation and proliferation by destabilizing the N-Myc oncoprotein. Nat Cell Biol. (2008) 10:643–53. doi: 10.1038/ncb1727 PMC2680438 18488021

[33] Zhong Q Gao W Du F Wang X. Mule/ARF-BP1, a BH3-only E3 ubiquitin ligase, catalyzes the polyubiquitination of Mcl-1 and regulates apoptosis. Cell. (2005) 121:1085–95. doi: 10.1016/j.cell.2005.06.009 15989957

[34] Hall JR Kow E Nevis KR Lu CK Luce KS Zhong Q. Cdc6 stability is regulated by the Huwe1 ubiquitin ligase after DNA damage. Mol Biol Cell. (2007) 18:3340–50. doi: 10.1091/mbc.e07-02-0173 PMC1951745 17567951

[35] Aravind L. The WWE domain: a common interaction module in protein ubiquitination and ADP ribosylation. Trends Biochem Sci. (2001) 26:273–5. doi: 10.1016/S0968-0004(01)01787-X 11343911

[36] Wilkinson CR Seeger M Hartmann-Petersen R Stone M Wallace M Semple C. Proteins containing the UBA domain are able to bind to multi-ubiquitin chains. Nat Cell Biol. (2001) 3:939–43. doi: 10.1038/ncb1001-939 11584278

[37] Zhou P Yang XL Wang XG Hu B Zhang L Zhang W. A pneumonia outbreak associated with a new coronavirus of probable bat origin. Nature. (2020) 579:270–3. doi: 10.1038/s41586-020-2012-7 PMC7095418 32015507

[38] Sokol CL Luster AD. The chemokine system in innate immunity. Cold Spring Harb Perspect Biol. (2015) 7:a016303. doi: 10.1101/cshperspect.a016303 25635046 PMC4448619

[39] Kotenko SV Gallagher G Baurin VV Lewis-Antes A Shen M Shah NK. IFN-lambdas mediate antiviral protection through a distinct class II cytokine receptor complex. Nat Immunol. (2003) 4:69–77. doi: 10.1038/ni875 12483210

[40] McNab F Mayer-Barber K Sher A Wack A A. Type I interferons in infectious disease. Nat Rev Immunol. (2015) 15:87–103. doi: 10.1038/nri3787 25614319 PMC7162685

[41] Tamura T Yanai H Savitsky D Taniguchi T. The IRF family transcription factors in immunity and oncogenesis. Annu Rev Immunol. (2008) 26:535–84. doi: 10.1146/annurev.immunol.26.021607.090400 18303999

[42] Ning S Pagano JS Barber GN. IRF7: activation, regulation, modification and function. Genes Immun. (2011) 12:399–414. doi: 10.1038/gene.2011.21 21490621 PMC4437765

[43] Loo YM Gale M Jr. Immune signaling by RIG-I-like receptors. Immunity. (2011) 34:680–92. doi: 10.1016/j.immuni.2011.05.003 PMC3177755 21616437

[44] Meraz MA White JM Sheehan KCF Bach EA Rodig SJ Dighe AS. Targeted disruption of the stat1 gene in mice reveals unexpected physiologic specificity in the JAK–STAT signaling pathway. Cell. (1996) 84:431–42. doi: 10.1016/S0092-8674(00)81288-X 8608597

[45] Flynn RA Belk JA Qi Y Yasumoto Y Wei J Alfajaro MM. Discovery and functional interrogation of SARS-CoV-2 RNA-host protein interactions. Cell. (2021) 184:2394–2411.e16. doi: 10.1016/j.cell.2021.03.012 33743211 PMC7951565

[46] Lahaye X Gentili M Silvin A Conrad C Picard L Jouve M. NONO detects the nuclear HIV capsid to promote cGAS-mediated innate immune activation. Cell. (2018) 175:488–501.e22. doi: 10.1016/j.cell.2018.08.062 30270045

[47] Villar J Cros A Juan De A Alaoui L Bonte PE Lau CM. ETV3 and ETV6 enable monocyte differentiation into dendritic cells by repressing macrophage fate commitment. Nat Immunol. (2023) 24:84–95. doi: 10.1038/s41590-022-01374-0 36543959 PMC9810530

[48] Hasan M Koch J Rakheja D Pattnaik AK Brugarolas J Dozmorov I. Trex1 regulates lysosomal biogenesis and interferon-independent activation of antiviral genes. Nat Immunol. (2013) 14:61–71. doi: 10.1038/ni.2475 23160154 PMC3522772

[49] Adhikary S Eilers M. Transcriptional regulation and transformation by Myc proteins. Nat Rev Mol Cell Biol. (2005) 6:635–45. doi: 10.1038/nrm1703 16064138

[50] Staller P Peukert K Kiermaier A Seoane J Lukas J Karsunky H. Repression of p15INK4b expression by Myc through association with Miz-1. Nat Cell Biol. (2001) 3:392–9. doi: 10.1038/35070076 11283613

[51] Phan RT Saito M Basso K Niu H Dalla-Favera R BCL6 interacts with the transcription factor Miz-1 to suppress the cyclin-dependent kinase inhibitor p21 and cell cycle arrest in germinal center B cells. Nat Immunol. (2005) 6:1054–60. doi: 10.1038/ni1245 16142238

[52] Yang J Hou C Wang H Perez EA Do-Umehara HC Dong H. Miz1 promotes KRAS-driven lung tumorigenesis by repressing the protocadherin Pcdh10. Cancer Lett. (2023) 555:216025. doi: 10.1016/j.canlet.2022.216025 36538983 PMC9870713

[53] Yang J Perez EA Hou C Zhang P Scoyk Van M Winn RA. Identification of the SARS-coV-2 entry receptor ACE2 as a direct target for transcriptional repression by miz1. Front Immunol. (2021) 12:648815. doi: 10.3389/fimmu.2021.648815 34305888 PMC8292894

[54] Barski A Cuddapah S Cui K Roh TY Schones DE Wang Z. High-resolution profiling of histone methylations in the human genome. Cell. (2007) 129:823–37. doi: 10.1016/j.cell.2007.05.009 17512414

[55] Rada-Iglesias A Bajpai R Swigut T Brugmann SA Flynn RA Wysocka J. A unique chromatin signature uncovers early developmental enhancers in humans. Nature. (2011) 470:279–83. doi: 10.1038/nature09692 PMC4445674 21160473

[56] Creyghton MP Cheng AW Welstead GG Kooistra T Carey BW Steine EJ. Histone H3K27ac separates active from poised enhancers and predicts developmental state. Proc Natl Acad Sci U.S.A. (2010) 107:21931–6. doi: 10.1073/pnas.1016071107 PMC3003124 21106759

