# Supplementary material for: Suppression of interferon α and γ response by Huwe1-mediated Miz1 degradation promotes SARS-CoV-2 replication
Source: Front Immunol. 2024 Jul 5;15:1388517. doi: 10.3389/fimmu.2024.1388517 (PMC11257858; doi:10.3389/fimmu.2024.1388517)
Supplement: Supplementary file 1 [file DataSheet_1.pdf]

Suppression of Interferon α and γ Response by Huwe1-Mediated Miz1 Degradation Promotes SARS-CoV-2 Replication

Vinothini Arunagiri[1,†], Laura Cooper[2,†], Huali Dong[1], Jake Class[2], Indrani Biswas[1], Sujan Vahora[1], Riddhi Deshpande[1], Khushi H Gopani[1], Guochang Hu[3], Justin M Richner[2], Lijun Rong[2,*], & Jing Liu[1,*]

Supplementary Figures

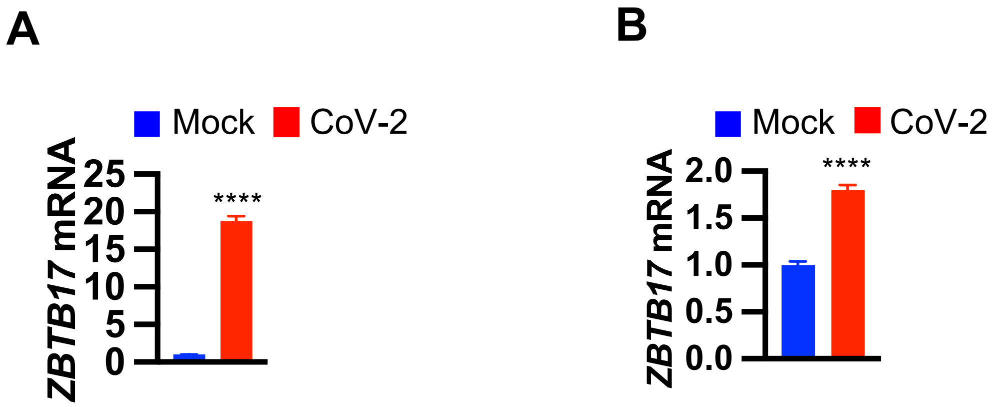

Supplementary Figure 1. Miz1 mRNA expression is upregulated following SARS-CoV-2 infection. Miz1 mRNA levels in mock- or SARS-CoV-2-infected A549/hACE2 (**A**) or Vero E6 cells (**B**) at 24 h post infection. Values represent the mean ± SEM. n=3. Unpaired Student's *t*-test was used. ****$p<0.0001$.

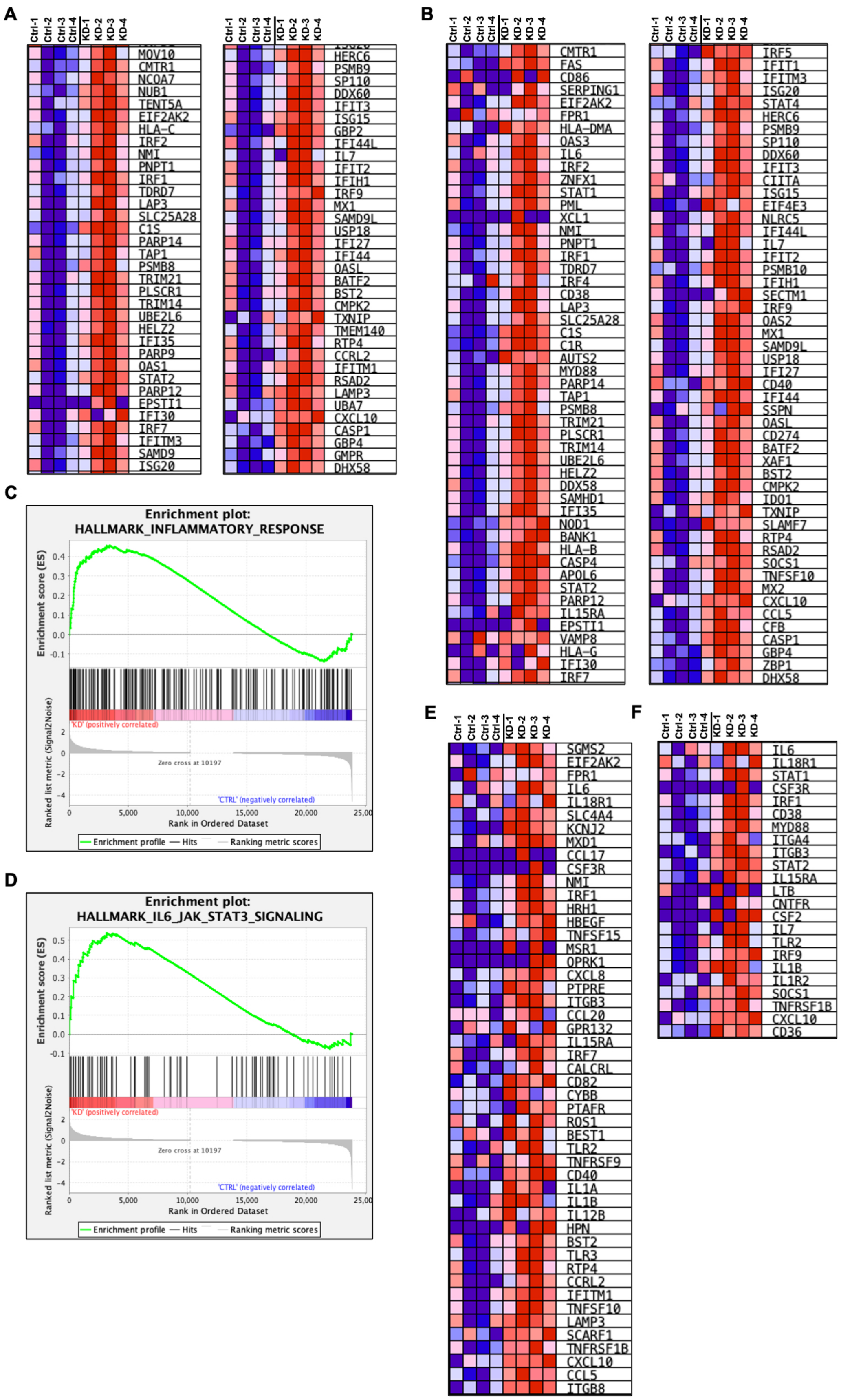
A
Ctrl-1
Ctrl-2
Ctrl-3
Ctrl-4
KD-1
KD-2
KD-3
KD-4
MOV10
CMTR1
NCOA7
NUB1
TENT5A
EIF2AK2
HLA-C
IRF2
NMI
PNPT1
IRF1
TDRD7
LAP3
SLC25A28
C1S
PARP14
TAP1
PSMB8
TRIM21
PLSCR1
TRIM14
UBE2L6
HELZ2
IFI35
PARP9
OAS1
STAT2
PARP12
EPSTI1
IFI30
IRF7
IFITM3
SAMD9
ISG20
HERC6
PSMB9
SP110
DDX60
IFIT3
ISG15
GBP2
IFI44L
IL7
IFIT2
IFIH1
IRF9
MX1
SAMD9L
USP18
IFI27
IFI44
OASL
BATF2
BST2
CMPK2
TXNIP
TMEM140
RTP4
CCRL2
IFITM1
RSAD2
LAMP3
UBA7
CXCL10
CASP1
GBP4
GMPR
DHX58
B
CMTR1
FAS
CD86
SERPING1
EIF2AK2
FPR1
HLA-DMA
OAS3
IL6
IRF2
ZNFX1
STAT1
PML
XCL1
NMI
PNPT1
IRF1
TDRD7
IRF4
CD38
LAP3
SLC25A28
C1S
C1R
AUTS2
MYD88
PARP14
TAP1
PSMB8
TRIM21
PLSCR1
TRIM14
UBE2L6
HELZ2
DDX58
SAMHD1
IFI35
NOD1
BANK1
HLA-B
CASP4
APOL6
STAT2
PARP12
IL15RA
EPSTI1
VAMP8
HLA-G
IFI30
IRF7
IRF5
IFIT1
IFITM3
ISG20
STAT4
HERC6
PSMB9
SP110
DDX60
IFIT3
CIITA
ISG15
EIF4E3
NLRC5
IFI44L
IL7
IFIT2
PSMB10
IFIH1
SECTM1
IRF9
OAS2
MX1
SAMD9L
USP18
IFI27
CD40
IFI44
SSPN
OASL
CD274
BATF2
XAF1
BST2
CMPK2
IDO1
TXNIP
SLAMF7
RTP4
RSAD2
SOCS1
TNFSF10
MX2
CXCL10
CCL5
CFB
CASP1
GBP4
ZBP1
DHX58
C
Enrichment plot:
HALLMARK_INFLAMMATORY_RESPONSE
Enrichment score (ES)
'KD' (positively correlated)
Zero cross at 10197
'CTRL' (negatively correlated)
Ranked list metric (Signal2Noise)
Rank in Ordered Dataset
Enrichment profile — Hits — Ranking metric scores
D
Enrichment plot:
HALLMARK_IL6_JAK_STAT3_SIGNALING
Enrichment score (ES)
'KD' (positively correlated)
Zero cross at 10197
'CTRL' (negatively correlated)
Ranked list metric (Signal2Noise)
Rank in Ordered Dataset
Enrichment profile — Hits — Ranking metric scores
E
SGMS2
EIF2AK2
FPR1
IL6
IL18R1
SLC4A4
KCNJ2
MXD1
CCL17
CSF3R
NMI
IRF1
HRH1
HBEGF
TNFSF15
MSR1
OPRK1
CXCL8
PTPRE
ITGB3
CCL20
GPR132
IL15RA
IRF7
CALCRL
CD82
CYBB
PTAFR
ROS1
BEST1
TLR2
TNFRSF9
CD40
IL1A
IL1B
IL12B
HPN
BST2
TLR3
RTP4
CCRL2
IFITM1
TNFSF10
LAMP3
SCARF1
TNFRSF1B
CXCL10
CCL5
ITGB8
F
IL6
IL18R1
STAT1
CSF3R
IRF1
CD38
MYD88
ITGA4
ITGB3
STAT2
IL15RA
LTB
CNTFR
CSF2
IL7
TLR2
IRF9
IL1B
IL1R2
SOCS1
TNFRSF1B
CXCL10
CD36

Supplementary Figure 2. Silencing Huwe1 augments interferon signaling during SARS-CoV-2 infection. (**A,B,E,F**) Heatmaps of representative genes from gene sets of "Interferon alpha response" (**A**), "Interferon gamma response" (**B**), "Inflammatory response" (**E**), and "IL6" (**F**) by Huwe1 KD in SARS-CoV-2-infected A549/hACE2 cells at 24 h post-infection, as analyzed by GSEA. (**C,D**) GSEA showing enrichment plots of the gene sets of "Inflammatory response" (**C**) and "IL6" (**D**) by Huwe1 KD in SARS-CoV-2 infected A549/hACE2 cells at 24 h post-infection.

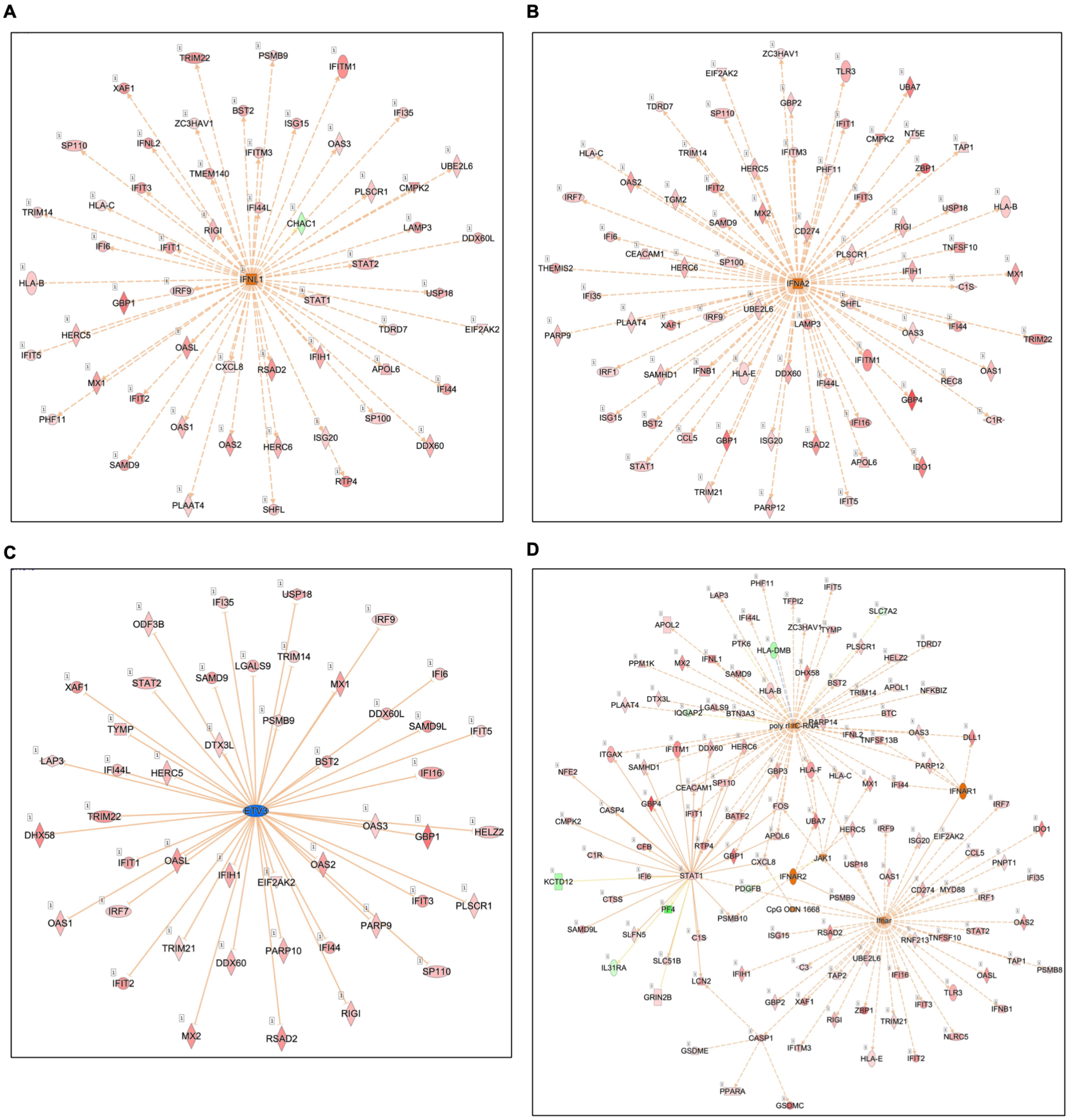

Supplementary Figure 3. Silencing Huwe1 enhances the antiviral response during SARS-CoV-2 infection. Causal Network Analysis in IPA identified significant regulatory nodes by Huwe1 KD in A549/hACE2 cells at 24 h post SARS-CoV-2 infection, including (**A**) IFNL1, (**B**) IFNA2, (**C**) ETV3, and (**D**) IFNAR gene cluster.

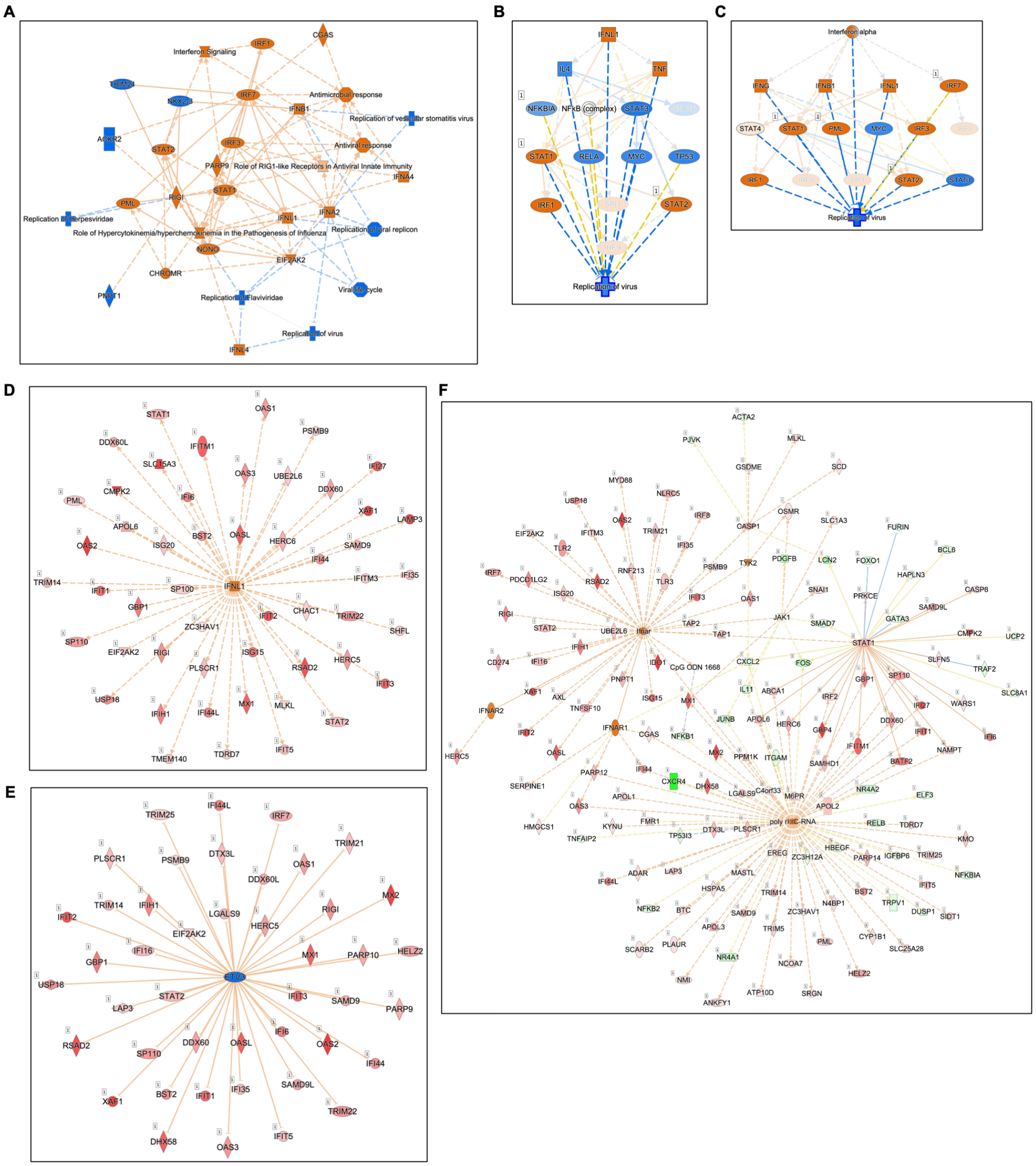

Supplementary Figure 4. Inhibition of Huwe1 with BI8622 recapitulates the effects of HUWE1 silencing on interferon signaling and antiviral response during SARS-CoV-2 infection. (**A**) A visual representation of the most significant canonical pathways and biological networks affected by Huwe1 inhibition with BI8622 in SARS-CoV-2 infected A549/hACE2 cells at 24 h post-infection using IPA core analysis, which revealed

interferon signaling and antiviral response. IPA upstream analysis revealed (**B**) IFNL1 and (**C**) Interferon alpha gene cluster among the top upstream regulators responsible for the gene expression changes resulting from inhibition of Huwe1 with BI8622 in SARS-CoV-2 infected A549/hACE2 cells at 24 h post-infection. Causal Network Analysis in IPA identified several important regulatory nodes in the resulting causal networks, including (**D**) IFNL1, (**E**) ETV3, and (**F**) IFNAR gene cluster.

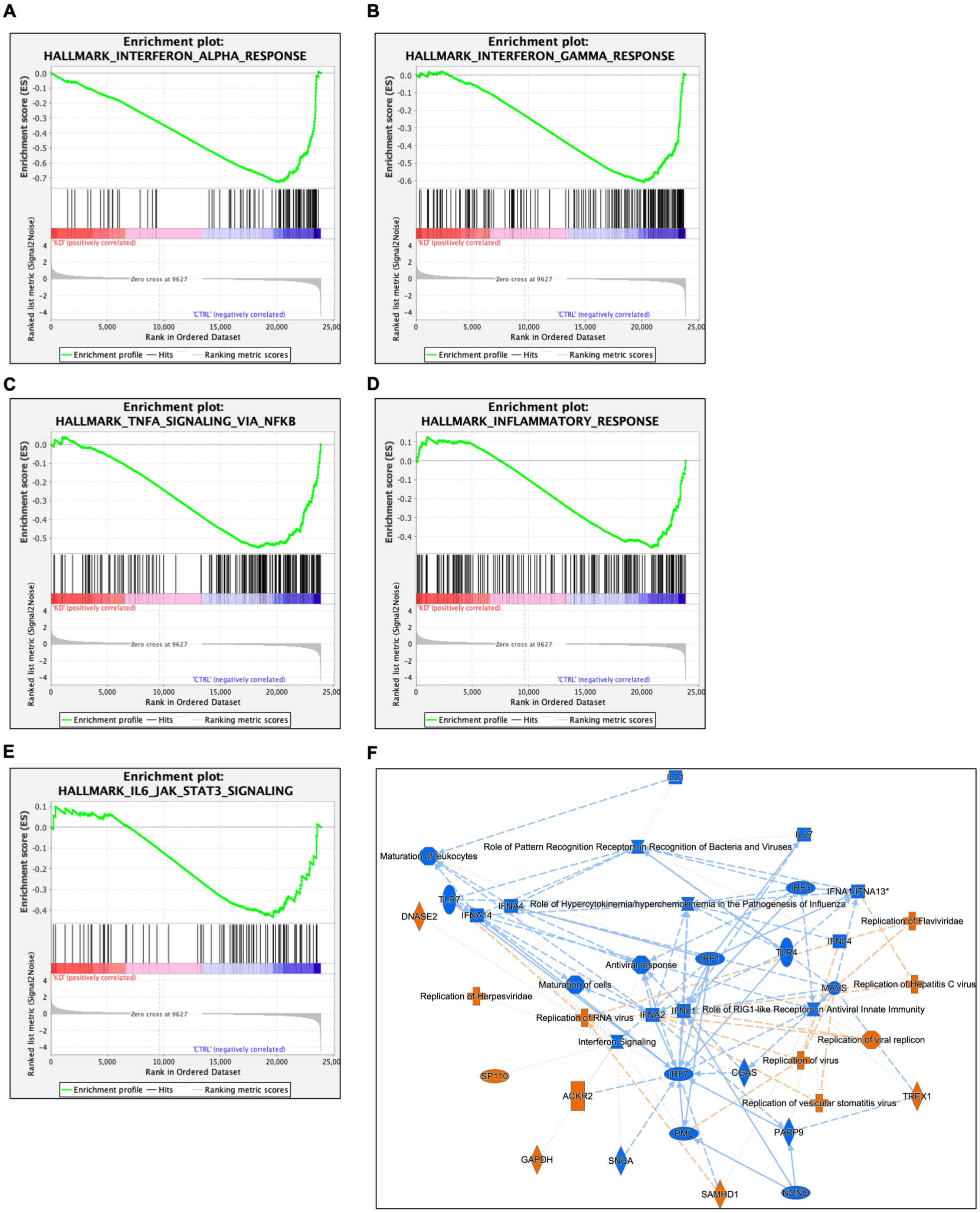

Supplementary Figure 5. Miz1 silencing suppresses interferon response during SARS-CoV-2 infection. (A-E) GSEA showing downregulated enrichment plots of the gene sets of "Interferon alpha response", "Interferon gamma response", "TNF signaling via NF-κB", "Inflammatory response", and "IL6 signaling" in SARS-CoV-2-infected

A549/hACE2 cells with Miz1 KD at 24 h post infection. (**F**) A visual representation of the most significant canonical pathways and biological networks by Miz1 KD in SARS-CoV-2-infected A549/hACE2 cells at 24 h post infection using IPA core analysis, revealing inhibited interferon signaling and antiviral response.

Supplementary Table

Upstream Regulator	Molecule Type	Predicted Activation State	Activation z-score	p-value of overlap	Mechanistic Network
IFNL1	cytokine	Activated	7.125	5.60E-69	263 (16)
TREX1	enzyme	Inhibited	-6.205	1.19E-63	185 (17)
ETV3	transcription regulator	Inhibited	-6.782	1.27E-62	
Interferon alpha	group	Activated	7.949	1.68E-60	240 (16)
IFNA2	cytokine	Activated	8.314	2.18E-60	232 (16)
IRGM	enzyme	Inhibited	-7.125	4.38E-60	169 (9)
IRF7	transcription regulator	Activated	7.797	7.49E-58	197 (16)
STAT1	transcription regulator	Activated	7.018	1.15E-57	248 (18)
ETV6	transcription regulator	Inhibited	-6.608	2.46E-56	207 (7)
NONO	transcription regulator	Activated	7.247	1.52E-54	83 (3)

Supplementary Table 1. Top ten upstream regulators identified by IPA Upstream Regulator Analysis in SARS-CoV-2 infected A549/hACE2 cells following Huwe1 KD.

Master Regulator	Molecule Type	Participating regulators	Predicted Activation	Activation z-score	p-value of overlap	Network bias-corrected p-value	Causal network
IFNL1	cytokine	IFNL1	Activated	7.147	7.63E-69	1.00E-04	55 (1)
IFNA2	cytokine	IFNA2	Activated	8.544	6.65E-66	1.00E-04	73 (1)
ETV3	transcription regulator	ETV3	Inhibited	-6.782	2.85E-65	1.00E-04	46 (1)
IRGM	enzyme	IRGM	Inhibited	-7.211	4.38E-60	1.00E-04	52 (1)
Ifnar	group	CASP1,CpG ODN 1668,Ifnar,IFNAR1,IFNAR2,JAK1,poly rI:rC-RNA,STAT1	Activated	9.545	5.50E-58	1.00E-04	121 (8)
IRF7	transcription regulator	IRF7	Activated	7.937	1.43E-57	1.00E-04	63 (1)
ETV6	transcription regulator	ETV6	Inhibited	-6.633	2.43E-57	1.00E-04	44 (1)
CGAS	enzyme	CGAS,IFN type 1,IRF3,STAT1,STING1,TBK1	Activated	9.037	1.31E-56	1.00E-04	97 (6)
mir-183	microRNA	IRF3,mir-183,STAT1	Activated	8.701	1.42E-56	1.00E-04	91 (3)
NONO	transcription regulator	NONO	Activated	7.285	5.59E-55	1.00E-04	57 (1)

Supplementary Table 2. Top ten master regulators identified by IPA Causal Network Analysis in SARS-CoV-2 infected A549/hACE2 cells following Huwe1 KD.

ID	Genes in dataset	Prediction (based on measurement direction)	Expr Log Ratio	Findings
ENSG00000182179	UBA7	Decreased	1.987	Decreases (2)
ENSG00000182393	IFNL1	Decreased	1.894	Decreases (2)
ENSG00000185885	IFITM1	Decreased	1.7	Decreases (1)
ENSG00000183709	IFNL2	Decreased	1.539	Decreases (2)
ENSG00000137752	CASP1	Decreased	1.315	Decreases (2)
ENSG00000115267	IFIH1	Decreased	1.308	Decreases (5)
ENSG00000184979	USP18	Increased	1.243	Increases (1)
ENSG00000164342	TLR3	Decreased	1.235	Decreases (3)
ENSG00000171855	IFNB1	Decreased	1.208	Decreases (3)
ENSG00000120217	CD274	Increased	1.196	Increases (1)
ENSG00000079385	CEACAM1	Affected	1.036	Affects (7)
ENSG00000173193	PARP14	Decreased	1.017	Decreases (1)
ENSG00000101347	SAMHD1	Increased	0.992	Increases (5)
ENSG00000059378	PARP12	Decreased	0.945	Decreases (1)
ENSG00000142089	IFITM3	Decreased	0.922	Decreases (1)
ENSG00000115415	STAT1	Decreased	0.832	Decreases (1)
ENSG00000196954	CASP4	Decreased	0.703	Decreases (2)
ENSG00000169871	TRIM56	Decreased	0.581	Decreases (4)
ENSG00000026559	KCNG1	Decreased	-0.888	Increases (1)

Supplementary Table 3. List of molecules involved in the decreased coronavirus replication function revealed by IPA Diseases and Functions Analysis following Huwe1 KD in SARS-CoV-2 infected A549/hACE2 cells.

Upstream Regulator	Molecule Type	Predicted Activation State	Activation z-score	p-value of overlap	Mechanistic Network
CHROMR	other	Activated	7.341	9.21E-56	
IFNL1	cytokine	Activated	7.009	3.88E-52	439 (15)
IRGM	enzyme	Inhibited	-6.278	1.21E-50	241 (8)
TREX1	enzyme	Inhibited	-5.884	4.68E-50	229 (12)
NONO	transcription regulator	Activated	7.574	2.32E-49	126 (4)
CSF1	cytokine		0.707	3.66E-49	358 (18)
ETV3	transcription regulator	Inhibited	-6.633	4.67E-49	
Interferon alpha	group	Activated	7.118	1.46E-46	317 (16)
ETV6	transcription regulator	Inhibited	-6.532	2.82E-45	336 (7)
TNF	cytokine	Activated	2.591	6.30E-45	467 (19)

Supplementary Table 4. Top ten upstream regulators by inhibition of Huwe1 with BI8622 in SARS-CoV-2 infected A549/hACE2 cells by IPA Upstream Regulator Analysis.

Master Regulator	Molecule Type	Participating regulators	Predicted Activation	Activation z-score	p-value of overlap	Network bias-corrected p-value	Causal network
CHROMR	other	CHROMR	Activated	7.344	9.21E-56	1.00E-04	69 (1)
ILRUN	other	AHR,Akt,Ap1,ARNT,carbon monoxide,CDX2,CTSB,HDAC3,HMOX1,IFNA1/IFNA13,IFNA10,IFNA14,IFNA16,IFNA17,IFNA2,IFNA21,IFNA4,IFNA5,IFNA6,IFNA7,IFNA8,IFNB1,IFNE,IFNK,IFNW1,ILRUN,IRF3,IRF7,JUN,MAPK8,NCOR-LXR-Oxysterol-RXR-9 cis RA,NFkB (complex),NFKBIA,NR3C1,RELA,RXRA,SMAD2,SMAD3,STAT1,STAT5a/b,YAP1	Inhibited	-5.416	2.46E-55	1.00E-04	264 (41)
Ifnar	group	CASP1,CpG ODN 1668,Ifnar,IFNAR1,IFNAR2,JAK1,poly rI:rC-RNA,STAT1,TYK2	Activated	7.234	1.14E-54	1.00E-04	148 (9)
IFN Lambda	group	Akt,ERK1/2,IFN Lambda,IFNL1,IFNL2,IFNL3,IFNL4,IFNLR1,JAK1,Mapk,STAT,Stat dimer,STAT1,STAT3,STAT4,STAT5A,STAT5a/b,STAT5B,STAT6,TYK2	Activated	4.454	1.60E-54	1.00E-04	213 (20)
MFN2	enzyme	ATF6,BAK1,BAX,EIF2AK3,ERK1/2,Jnk,MAPK1,MFN2,RAF1	Activated	3.266	7.13E-54	1.00E-04	150 (9)
Interferon alpha	group	Ap1,BAK1,BAX,CASP8,CCND1,CCNE1,CD247,Cdk,CDK2,DAXX,EIF4E,ERBB3,ERK1/2,IFNA2,IFNA4,Ifna4,Ifnar,IFNAR1,Interferon alpha,IRF1,IRF3,JAK1,Jnk,MAP3K1,MAPK1,MAPKAPK2,MAPKAPK3,MKNK1,MMP9,MTOR,MTORC1,NFkB (complex),NR3C1,P38 MAPK,p70 S6k,Pkc(s),PTPN1,RAC1,RB1,resiquimod,STAT,STAT1,STAT3,STAT4,STAT5A,STAT5a/b,STAT5B,STAT6,TERT,TYK2,ZAP70	Activated	5.677	1.76E-52	1.00E-04	298 (51)
ETV3	transcription regulator	ETV3	Inhibited	-6.633	2.02E-51	1.00E-04	44 (1)
IRGM	enzyme	IRGM	Inhibited	-6.181	1.21E-50	1.00E-04	53 (1)
IFNL1	cytokine	IFNL1	Activated	7.141	1.74E-50	1.00E-04	51 (1)
NONO	transcription regulator	NONO	Activated	7.62	7.70E-50	1.00E-04	62 (1)

Supplementary Table 5. Top ten upstream regulators by inhibition of Huwe1 with BI8622 in SARS-CoV-2 infected A549/hACE2 cells identified by IPA Upstream Regulator Analysis.

ID	Genes in dataset	Prediction (based on measurement direction)	Expr Log Ratio	Findings
ENSG00000183486	MX2	Decreased	2.967	Decreases (1)
ENSG00000165949	IFI27	Decreased	2.79	Decreases (1)
ENSG00000134321	RSAD2	Decreased	2.743	Decreases (11)
ENSG00000135114	OASL	Decreased	2.52	Decreases (13)
ENSG00000108771	DHX58	Affected	2.491	Affects (1)
ENSG00000157601	MX1	Decreased	2.476	Decreases (12)
ENSG00000185885	IFITM1	Decreased	2.45	Decreases (13)
ENSG00000185745	IFIT1	Decreased	2.444	Decreases (4)
ENSG00000187608	ISG15	Decreased	2.321	Decreases (24)
ENSG00000126709	IFI6	Decreased	2.104	Decreases (1)
ENSG00000117228	GBP1	Decreased	2.08	Decreases (1)
ENSG00000117595	IRF6	Increased	2.056	Increases (1)
ENSG00000115267	IFIH1	Decreased	1.935	Decreases (19)
ENSG00000130303	BST2	Decreased	1.831	Decreases (4)
ENSG00000184979	USP18	Increased	1.8	Increases (1)
ENSG00000107201	RIGI	Decreased	1.728	Decreases (15)
ENSG00000111331	OAS3	Decreased	1.66	Decreases (2)
ENSG00000135899	SP110	Decreased	1.595	Decreases (1)
ENSG00000137462	TLR2	Increased	1.584	Increases (3)
ENSG00000089127	OAS1	Decreased	1.558	Decreases (3)
ENSG00000138646	HERC5	Decreased	1.535	Decreases (1)
ENSG00000121858	TNFSF10	Affected	1.514	Affects (4)
ENSG00000120217	CD274	Increased	1.396	Increases (1)
ENSG00000185507	IRF7	Increased	1.392	Increases (1)
ENSG00000140853	NLRC5	Decreased	1.35	Decreases (2)
ENSG00000137752	CASP1	Decreased	1.333	Decreases (2)
ENSG00000133083	DCLK1	Increased	1.302	Increases (1)
ENSG00000168961	LGALS9	Decreased	1.219	Decreases (2)
ENSG00000173193	PARP14	Decreased	1.21	Decreases (1)
ENSG00000115415	STAT1	Decreased	1.209	Decreases (9)
ENSG00000101347	SAMHD1	Increased	1.134	Increases (6)
ENSG00000106785	TRIM14	Increased	1.116	Increases (1)
ENSG00000059378	PARP12	Decreased	1.063	Decreases (5)
ENSG00000168394	TAP1	Increased	1.058	Increases (4)
ENSG00000170581	STAT2	Decreased	1.048	Decreases (1)
ENSG00000132109	TRIM21	Increased	0.981	Increases (1)
ENSG00000164342	TLR3	Decreased	0.962	Decreases (7)
ENSG00000172183	ISG20	Decreased	0.933	Decreases (3)
ENSG00000187688	TRPV2	Increased	0.907	Increases (1)
ENSG00000055332	EIF2AK2	Decreased	0.906	Decreases (22)
ENSG00000140464	PML	Decreased	0.822	Decreases (14)
ENSG00000156587	UBE2L6	Decreased	0.817	Decreases (1)
ENSG00000121060	TRIM25	Decreased	0.8	Decreases (3)
ENSG00000124201	ZNFX1	Affected	0.799	Affects (1)
ENSG00000142089	IFITM3	Decreased	0.785	Decreases (18)
ENSG00000067066	SP100	Decreased	0.775	Decreases (2)
ENSG00000124614	RPS10	Increased	0.701	Increases (1)
ENSG00000196116	TDRD7	Decreased	0.697	Decreases (1)
ENSG00000132256	TRIM5	Decreased	0.684	Decreases (5)
ENSG00000172936	MYD88	Decreased	0.652	Decreases (12)

Supplementary Table 6. List of molecules involved in the decreased virus replication function revealed by IPA Diseases and Functions Analysis following inhibition of Huwe1 with BI8622 in SARS-CoV-2 infected A549/hACE2 cells.

Huwe1-5': 5'-TGC TGA AGT GTC ACC CAC AG-3'

Huwe1-3': 5'-CCC AGC TCC CAC TAT AAC CTC-3'

Miz1-5': 5'-GGG CAG GTG CTG GAG TTT AT-3'

Miz1-3':5'-AAC AGG GCA GAC CTT CTG TG-3'

Supplementary Table 7. Primer sequences for qRT-PCR analysis of Huwe1 and Miz1 genes.

Ifna1-5’: 5’- TCA CGG TGG GTT CAA TTA GGA-3’

Ifna1-3’: 5’- AAT GCT TCT GGG CCA CTT TC -3’

Ifna2-5’: 5”- ACA CAG GGG CAT TTG GAA AA -3’

Ifna2-3’: 5’- GCC ACC AGT AAA GCA AAG GT -3’

Supplementary Table 8. Primer sequences for ChIP-qPCR.
